# Identification of Novel Marker–Trait Associations for Lint Yield Contributing Traits in Upland Cotton (*Gossypium hirsutum* L.) Using SSRs

**DOI:** 10.3389/fpls.2021.653270

**Published:** 2021-05-26

**Authors:** Pawan Kumar, Somveer Nimbal, Rajvir Singh Sangwan, Neeraj Budhlakoti, Varsha Singh, Dwijesh Chandra Mishra, Sagar, Raju Ram Choudhary

**Affiliations:** ^1^Department of Genetics and Plant Breeding, CCS Haryana Agricultural University, Hisar, India; ^2^Indian Council of Agricultural Research-Indian Agricultural Statistics Research Institute, New Delhi, India; ^3^Department of Molecular Biology and Biotechnology, CCS Haryana Agricultural University, Hisar, India

**Keywords:** association mapping, *Gossypium hirsutum*, lint yield, linkage disequilibrium, SSR, CMLM, MLMM

## Abstract

Improving the yield of lint is the main objective for most of the cotton crop improvement programs throughout the world as it meets the demand of fiber for textile industries. In the current study, 96 genotypes of *Gossypium hirsutum* were used to find novel simple sequence repeat marker-based associations for lint yield contributing traits by linkage disequilibrium. Extensive phenotyping of 96 genotypes for various agronomic traits was done for two consecutive years (2018 and 2019) in early, normal, and late sown environments. Out of 168 SSR markers screened over the 96 genotypes, a total of 97 polymorphic markers containing 293 alleles were used for analysis. Three different models, i.e., mixed linear model (MLM), compressed mixed linear model (CMLM), and multiple locus mixed linear model (MLMM), were used to detect the significant marker–trait associations for six different environments separately. A total of 38 significant marker–trait associations that were common to at least two environments were considered as promising associations and detailed annotation of the significant markers has been carried out. Twenty-two marker–trait associations were found to be novel in the current study. These results will be very useful for crop improvement programs using marker-assisted cotton breeding.

## Introduction

Cotton is the most important fiber crop in the world and comprises 52 different species. Among these, two diploid species (2n = 2x = 26), *viz. Gossypium arboreum* L. and *Gossypium herbaceum* L., and two tetraploids (2n = 4x = 52), *viz. Gossypium barbadense* L. and *Gossypium hirsutum* L., are commercially grown and considered to be very important. *G. hirsutum* L. itself covers ∼95% of the global land area under production of cotton because of its wider adaptability and high lint yield. India has the largest area and production for cotton in the world though productivity (yield per hectare) is considerably lesser as compared with the rest of the world (ICAR-AICRP, 2019–2020). To cope with the increasing demand for cotton in textile industries, it is very essential to develop high-yielding cotton varieties and hybrids. In the last few decades, most of the available germplasm has been used in conventional breeding based on morphological markers for the development of cotton hybrids or varieties (Van Esbroeck and Bowman, 1998). Morphological traits classify the genotypes only on the basis of visible variations that can be affected by environmental changes as well as agronomic practices. The development of hybrids and varieties for higher lint yield with desirable fiber quality parameters is the most important objective of the cotton improvement programs all over the world. However, further enhancing cotton productivity is a challenging task for breeders due to extensive use of locally available germplasm (Tyagi et al., 2014; Zhang et al., 2020) and the high impact of environmental fluctuations on these yield contributing traits.

In plants, molecular background for traits such as yield and quality is identified by quantitative trait locus (QTL) mapping, which includes high-density molecular marker linkage map constructions in a population developed using two or limited parents (Fang et al., 2013). Usage of bi-parental population for QTL mapping covers a small portion of the genome and is a costly, tedious, and higher risk task. It is a “classical approach” and will continue to be the main tool for gene tagging in crops; however, it is very expensive (Stich et al., 2006) and has low resolution while evaluating only a few alleles simultaneously (Flint-Garcia et al., 2003). A major drawback of QTL linkage mapping is that it is deficient in fine mapping, as only a few available meiotic events are used in the mapping (Jannink and Walsh, 2002). To overcome the drawback of bi-parental QTL mapping, LD (linkage disequilibrium)-based association mapping has attained popularity among plant geneticists in the last two decades (Jannink et al., 2001). The starting point for association mapping is based on the non-random associations of alleles present at different loci or marker locus to the phenotypic trait. LD-based association mapping uses natural populations for mapping purposes and thus it is a method with high resolution. However, LD can be caused by many underlying factors such as unknown population structure and several forces, which include mutation, genetic bottlenecks, drift, founder effects, selection, and inbreeding level (particularly for plants). Characterization of LD level and patterns in a population are pre-requisite for use of LD mapping in crop plants. Discrimination between physical LD and other forces that can create LD in natural populations is important to avoid the detection of spurious associations. LD decay with respect to an increase in distance between markers is faster in outcrossing plants than inbreeding plants (Zhao et al., 2014). A population-based association study has many advantages over bi-parental traditional QTL-mapping such as (a) many alleles are evaluated simultaneously, so there are broader genetic variations available with wider background for the marker–trait association (MTA); (b) higher mapping resolution due to the number of recombination events that occurred over a long germplasm development history; and (c) the process is also time-saving and cost-effective (Hansen et al., 2001; Kraakman et al., 2004, 2006).

In cotton, the first attempt at association mapping was taken by Kantartzi and Stewart in 2008. In this study, they detected 30 markers associated with fiber-related traits based on 98 simple sequence repeat (SSR) markers using 56 *G. arboreum* genotypes. Afterward, a number of attempts have been made even using next-generation sequencing (NGS) to find the MTA for various agronomic traits (Cai et al., 2014; Mei et al., 2017; Dong C. et al., 2018; Ali et al., 2020; Zhang et al., 2020), fiber quality traits (Nie et al., 2016; Abdullaev et al., 2017; Ademe et al., 2017; Iqbal and Rahman, 2017; Dong C. G. et al., 2018; Huang et al., 2018), and biotic/abiotic stress tolerance (Wang et al., 2016; Zhao et al., 2016; Baytar et al., 2017; Sun et al., 2019) using molecular marker-based approach exhausting different germplasm of *Gossypium* sp. Though association studies based on SNP through genotyping by sequencing (GBS) and NGS are more promising and more efficient than SSR, the cost of sequencing makes this technique not readily available for researchers in undeveloped and developing countries, while easy accessibility of PCR thermo-cycler and SSR markers has kept their importance in the research field. Application of LD-based association mapping in cotton has not only accelerated MAS programs but also added to our knowledge and understanding of the complex cotton genome and its evolution. Most of the association studies in cotton were mainly focused on fiber quality and abiotic stress resistance. The present study is motivated to deciphering the various molecular marker loci associated with lint yield and its contributing traits using the association mapping approach. In the current study, we have utilized a large cotton germplasm resource from CCS HAU, Hisar, which is novel for association mapping study, to identify novel MTAs concerning lint yield contributing traits using association mapping strategy in different sown environments.

## Materials and Methods

### Germplasm Collection

The experimental material for the present study is composed of 96 genotypes of upland cotton (*G. hirsutum*) selected from breeding material collected from Cotton Section, the Department of Genetics and Plant Breeding, CCS HAU, Hisar (Supplementary Table 1).

### Phenotyping

A total of 96 germplasm lines of upland cotton (*G. hirsutum*) were grown in a randomized block design (RBD) with two replications in early (before April 26), normal (from April 26 to May 10), and late (after May 10) sown conditions in the experimental field of Cotton Section, the Department of Genetics and Plant Breeding, CCS HAU, Hisar, during *kharif* season of 2018 and 2019. The agronomic practices recommended for Haryana state were followed to raise a good crop. The fertilizer dose was applied at 86 kg nitrogen, 30 kg phosphorus, and 25 kg zinc sulfate per hectare. For control of weeds, the pre-emergence herbicide, Pendimethalin, at 5 L per hectare was applied and, subsequently, weeds were managed with hoeing by kasola and mechanical intercultural hoeing with power weeder. The data were recorded on five randomly selected plants of each genotype in each replication and for different underlying lint yield contributing traits, *viz.* days to first flower (DF), plant height in cm (PH), number of monopods per plant (NM), number of bolls per plant (NB), boll weight in grams (BW), seed cotton yield (SCY), lint yield (LY), ginning out turn in percent (GOT), seed index (SI), and lint index (LY).

### Statistical Analysis

Analysis of variance popularly known as ANOVA was carried out to determine genotype, year, genotype × year, and other higher interaction terms variances for different traits of cotton using SAS version 9.4 with PROC GLM statement (SAS Institute Inc., Cary, NC, United States). The relationship between yield and other yield attributing traits was further explored by correlation analysis. To have a better understanding of how phenotypic values of traits are distributed, box plots for different traits for underlying environments were generated. This analysis was carried out using R software (R Core Team, 2019).

### DNA Isolation

Genomic DNA was isolated from young leaves of 96 genotypes of cotton using standard protocol, i.e., using cetyl trimethyl ammonium-bromide (CTAB) procedure and following the approach of Saghai-Maroof et al. (1984). The quality of DNA samples was analyzed using 0.8% agarose gel electrophoresis. One hundred sixty-eight SSR markers (Supplementary Table 2) distributed over 26 linkage groups of A and D genome (of *G. hirsutum*) were used for molecular screening of the genotypes, out of which 97 markers showed polymorphism and the same has been further used for downstream analysis. The sequence of these primer pairs and details required for PCR (polymerase chain reaction) was obtained from the COTTONGEN resource^1^. The optimized PCR reaction mixture (10 μl) contained 50 ng of DNA template, 5 μl of DreamTaq Green PCR Master Mix (2×), 0.5 μM of primers, and 3 μl of nuclease-free water. PCR products were resolved by gel electrophoresis using 2.5% agarose gels (Sigma) at 4 V/cm in 1.0× TBE buffer. DNA banding patterns of 96 genotypes of cotton were observed under UV light with staining of electrophoretic gels in ethidium bromide (0.5 μg/ml). An amplified band at each position was scored as 1 for presence and 0 for absence. The size (in nucleotide base pairs) of the amplified bands was further determined based on its migration related to the standard 50-bp/100-bp DNA ladder.

### Scoring of SSR Markers and Assessment of Genetic Diversity

Molecular weights of SSR products (in bp) were estimated and preliminary statistical analysis of the genotypes was performed using POWERMARKER V 3.25 (Liu and Muse, 2005). The total number of alleles, major allele frequency, gene diversity, and polymorphism information content (PIC) values were calculated for each marker. Furthermore, the genetic diversity of a sub-population was identified using POPULATION STRUCTURE analysis.

### Population Structure Analysis

Analysis of the population structure of 96 upland cotton (*G. hirsutum* L.) genotypes was carried out using the software STRUCTURE V 2.3.4 (Pritchard et al., 2000). An admixture model was selected to estimate the number of sub-populations (i.e., *K* value) for studied genotypes. Initially, 10 runs for each value of *K* ranging from 1 to 10 were conducted with additional parameters of 10,000 burn-in length and number of replications. Finally, number of sub-populations (*K* value) was estimated by following the approach of Evanno et al. (2005) implemented in a web-based utility STRUCTURE HARVESTER (Earl, 2012), i.e., by plotting the distribution of Δ*K*, which is an ad hoc statistic based on the rate of change in the log probability of data between successive *K* values. The value of Δ*K* was calculated as a mean of absolute values of the difference between successive likelihood values of *K* divided by its standard deviation of *L*(*K*). The highest value obtained from the graph by plotting Δ*K* values most accurately detects the uppermost hierarchical level of structure. The Δ*K* plots for the studied genotypes were further generated using the STRUCTURE HARVESTER.

### Analysis of Molecular Variance (AMOVA) and Genetic Diversity

Analysis of molecular variance of a sub-population identified using POPULATION STRUCTURE analysis was assessed using GenAlex version 6.5 (Peakall and Smouse, 2006). The parameters computed in sub-populations for genetic diversity were the total number of alleles per locus (Na), number of effective alleles per locus (Ne), Shannon’s information index (I), observed gene diversity (h), and unbiased gene diversity (uh) following the protocol given by Nei and Li (1979).

### LD and Association Mapping

Linkage disequilibrium is the non-random co-segregation of alleles at two or more loci. This non-random co-segregation could be between loci on the same chromosome or between loci on different chromosomes. The tight linkage between two alleles on the same chromosome can be translated in high LD. Therefore, LD can be measured as allele frequency correlation (*r*^2^) between the pairs of markers located on the same chromosome. TASSEL software (Bradbury et al., 2007) was used to study LD.

For Association mapping, R package Genome Association and Prediction Integrated Tool (GAPIT) (Lipka et al., 2012) was used to conduct the analysis. Here, for GWAS analysis, different association models were tested separately for the cotton germplasm panel, i.e., (1) compressed mixed linear model (CMLM), (2) multiple locus mixed linear model (MLMM), and (3) mixed linear model (MLM). As additional information, marker-based kinship matrix (K) was also generated using marker genotype information based on the VanRaden method; the same was further used to generate a clustering heat map for marker panel using GAPIT. All the additional information like population structure (*Q*) and kinship information (K) were used as covariates while fitting GWAS models. Separate GWAS analysis was conducted for different environments under study using different models, i.e., MLMM, CMLM, and MLM, applied separately and presented the results. Moreover, to get a better representation of MTA, we have also highlighted those markers that were found to be significantly consistent to at least two environment models. A *p*-value ≤ 0.01 was used as a threshold to declare significant MTA. The same was represented using Manhattan plots and quantile–quantile plots (QQ plots) between observed and expected *p* values of association, which revealed the fitting of the model.

## Results

### Phenotyping

The results of ANOVA are presented in Table 1 which showed the mean sum of squares due to genotypes and date of sowing (DS) was highly significant (*p* < 0.0001) for all studied traits except NM. Mean sum of square due to interaction of genotype × year, genotype × DS, and year × DS was found to be highly significant for DF, PH, NM, NB, SI, SCY/P, SI, and LI (Table 1). Analysis of higher interaction terms, i.e., Genotype × Year × DS was also found to be significant for most of the traits except for BW and NM. These results indicate that adequate variability was present for lint yield and its contributing traits for the material used in the study. In order to better understand the distribution of phenotypic traits over the different environments, box plots of the same have been drawn (Supplementary Figure 1). Mean values for DF were reported to be highest in the early sown environment of the year 2019 (E19) followed by normally sown environment 2019 (N19), and early and normal sown environment of 2018 (E18 and N18), while the late sown environment of both years 2018 (L18) and 2019 (L19) had reported early flowering, i.e., least DF (Supplementary Figure 1a). The highest average of PH was recorded in the early sown-2018 (E18) trial, and the minimum average plant height was observed in late sown-2019 (L19) and late sown-2018 (L18) crop (Supplementary Figure 1b). Average BW was reported highest in early sown-2018 (E18) followed by normal sown-2018 (N18) and early sown-2019 (E19). However, the least average was observed in the late sown-2019 (L19) and late sown-2018 (L18) environment (Supplementary Figure 1c). Mean values for NB were reported highest in E18 followed by N18 and E19 environments, while the lowest value of mean for NB was reported in L18 and L19 environments of both years (Supplementary Figure 1d). Average NM was reported at par in all six environments (Supplementary Figure 1e). The highest average of GOT was observed in N18, followed by N19 and E18 environments. However, the lowest average of ginning out turn was reported in E19 and L18 environments (Supplementary Figure 1f). The highest average of seed index was reported in N18 while the lowest average was recorded in the L19 environment (Supplementary Figure 1g). The highest average of SCY/P was reported in N18, followed by E18 and N19 environments. However, the lowest average of SCY/P was reported in L19 and L18 environments (Supplementary Figure 1h). The highest average of LY was observed in normal sown-2018 (N18); the lowest average of LY was reported in late sown-2019 (L19) and late sown-2018 (L18) trials. However, SI and LI had manifested similar patterns among different environments (Supplementary Figures 1i,j). The highest average of SI and LI was rerecorded in the normal (timely) sown-2018 (N18) experiment while the lowest average for both the traits was recorded in the late sown-2019 (L19) environment.

**TABLE 1:** Analysis of variance for lint yield and its contributing traits.

Source	Df	DF	PH	NM	NB	BW	SCY/P	GOT	LY	SI	LI
Genotype	95	10.172*	1346.373*	1.264	203.098*	0.739*	2726.145*	12.252*	364.015*	2.289*	0.953*
Year	1	2538.281*	20,520.004*	7.508	536.281*	2.320*	19,692.855*	1.750	2691.945*	135.301*	45.311*
DS	2	4371.781*	84,070.831*	15.258*	15,087.553*	93.485*	491,300.264*	84.698*	65,206.867*	25.775*	12.296*
Rep	1	78.125*	4.014	0.043	11.281	0.005	1411.133	1.300	328.961*	28.943*	7.474*
Genotype × Year	95	3.815	1333.572*	1.252	20.380*	0.104	307.216*	13.659*	60.200*	2.751*	0.906*
Genotype × DS	190	3.815*	496.888	0.955	22.404*	0.052	361.379*	7.528*	56.603*	1.155*	0.657*
Year × DS	2	721.781*	5535.089*	17.518*	62.258*	0.002	1311.295*	18.245	80.964	30.847*	6.038*
Genotype × Year × DS	190	3.815*	566.403*	1.086	7.803*	0.032	114.775	8.466*	22.365	1.123*	0.619*

DS, date of sowing; Rep, replication; Df, degree of freedom; DF, days to first flower; PH, plant height; BW, boll weight; NB, number of boll per plant; NM, number of monopods per plant; GOT, ginning out turn; SI, seed index; SCY/P, seed cotton yield per plant; LY, lint yield; LI, lint index. *Significant at p < 0.0001.

**FIGURE 1:**
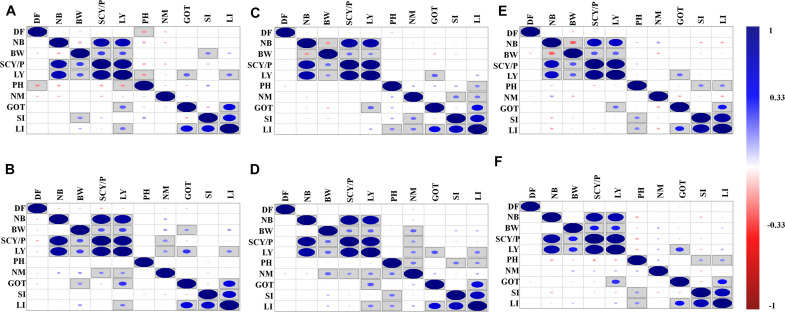
Phenotypic correlations of lint yield contributing traits over six different environments: **(A)** early sown-2018, **(B)** early sown-2019, **(C)** normal sown-2018, **(D)** normal sown-2019, **(E)** late sown-2018, and **(F)** late sown-2019. DF, days to flower; PH, plant height; BW, boll weight; NB, number of boll per plant; NM, number of monopods per plant; GOT, ginning out turn; SI, seed index; SCY/P, seed cotton yield per plant; LY, lint yield; LI, lint index. Here, in the figure upper diagonal and lower diagonal are the same blue and red color highlights for positive and negative correlation, respectively. Correlations significant at *p* < 0.05 are marked by square in the box. The size of the dots represents the strength (value) of correlation; i.e., the bigger the dots, the higher the value of correlation (toward +1 or -1 for blue and red color, respectively).

The correlation analysis for lint yield and contributing traits of cotton for different environments was also conducted, and results are shown in Figure 1. The environment-wise phenotypic correlation results showed that NB and BW had a strong and positive correlation with SCY and LY in all six environments (Figure 1). However, NM showed a positive and significant correlation with SCY and LY, only in early and normal sown environments of 2019. Boll weight showed a negative correlation with NB in all three environments of 2018, but a negative significant correlation was observed in normal and late planting environment of 2018. However, no significant correlation was observed for the same traits in 2019 in any of the sowing conditions. Ginning out turn recorded a significant positive correlation with lint yield in all the environments except the early sown environment of 2018.

### Molecular Diversity

A total of 97 polymorphic markers were used for the analysis of molecular diversity. All 97 polymorphic markers were distributed on 26 chromosomes with an average of 3.73 markers per chromosome. Among these, 47 (48%) were located on the 13 chromosomes of A-genome while the remaining 50 (52%) markers were distributed over D-genome. Chromosomes A9 and D9 had maximum (six) polymorphic markers while Chromosome A7 had only one polymorphic marker.

Ninety-seven polymorphic markers contained 293 different alleles ranging from two to five alleles per marker with an average value of 3.020 alleles per marker. Out of 97 markers, 39 markers amplified three polymorphic alleles while only four markers amplified five polymorphic alleles in the studied genotypes.

**FIGURE 2:**
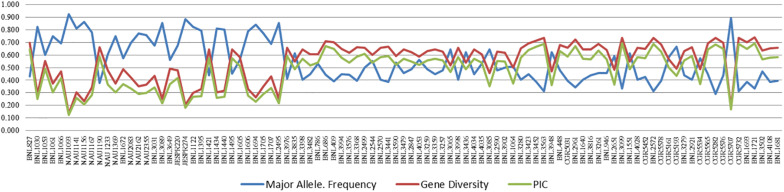
Variation in major allele frequency, gene diversity, and PIC for 97 polymorphic markers of cotton. A list of the markers on *X*-axis is also given in Supplementary Table 3.

Major allele frequency ranged from 0.292 (CGR5282) to 0.927 (NAU1093) with a high average value of 0.551. Gene diversity for SSRs ranged from 0.135 (NAU1093) to 0.740 (BNL1721) with an average value of 0.551. The PIC value for SSR markers ranged from 0.126 (NAU1093) to 0.693 (BNL1721), with an average value of 0.485. For better visualization, the pattern of variation of major allele frequency, gene diversity, and PIC is also represented graphically in Figure 2 and Supplementary Table 3.

### Population Structure

**FIGURE 3:**
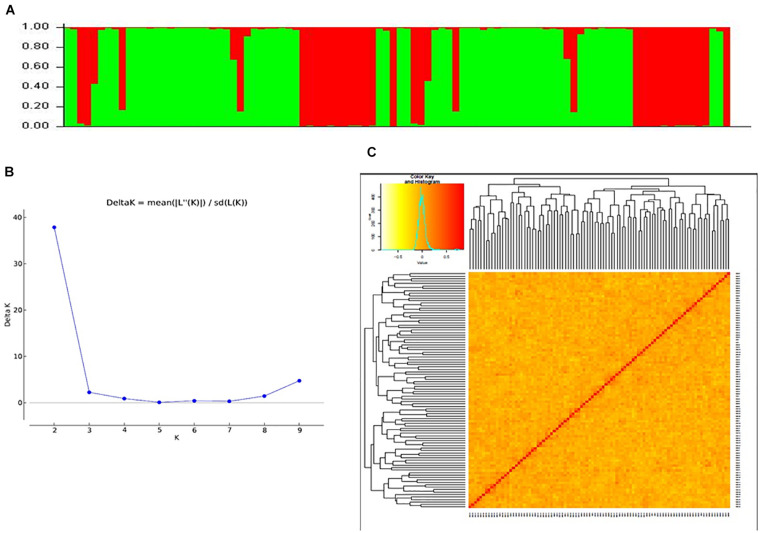
**(A)** Bar graph for population structure of cotton genotypes performed by admixture method in STRUCTURE, which grouped all accessions into two clusters. **(B)** Estimation of number of clusters using Δ*K* values for *K* ranging from 2 to 10. **(C)** Heat map plot displaying relationships of 96 cotton genotypes based on SSR markers.

Data generated from 97 SSR markers were subjected to STRUCTURE analysis to examine the presence of sub-groups if any. A cutoff of 70% membership probability was used as a threshold value for placing a genotype into a particular cluster using the admixture model. Results of STRUCTURE analysis conceded the presence of two major sub-groups in the populations (Figures 3A,B). These sub-groups contained 32 (red cluster) and 60 (green cluster) genotypes of cotton. Four genotypes could not match the cutoff membership probability of any of the clusters and were considered as a mixture. Most of the genotypes of an individual cluster had shown 100% membership probability. Clustering of cotton genotypes by population structure had been similar to the ancestral history of the genotypes. Results of population structure are supported by a heat map plot drawn by GAPIT to depict the level of genomic similarity among the genotypes (Figure 3C).

### Analysis of Molecular Variance

AMOVA was conducted using GenAlEx (v 6.5), which revealed that difference between groups obtained from STRUCTURE analysis was around 2% of the total variation contributed by the whole germplasm. However, 98% variation was attributed to diversity between individuals within a group (Table 2). Fixation index (FST) value for the whole population (0.019) was significant at *p* < 0. 001. Variation among genotypes of the whole population is significantly high though pairwise FST values revealed that variation among sub-populations is comparatively lower.

**TABLE 2:** Analysis of molecular variance for sub-groups estimated by structure analysis.

Source	Df	SS	MS	Est. Var.	%
**Among population**	1	49.578	49.578	0.532	2%
**Within population**	94	2526.266	26.875	26.875	98%
**Total**	95	2575.844		27.407	100%

*Df, degree of freedom; SS, sum of square; MS, mean sum of square; Est. Var., estimated variance; %, percent of variance.*

In the present study, the “Na” (number of different alleles per locus) was 2.887 and 2.990 for sub-populations 1 and 2 of cotton germplasm, respectively, with an average value of 2.938. In Na, 2.406 and 2.403 were Ne (number of effective alleles per locus) in each population, respectively, with a mean value of 2.404. While NP (number of private alleles per locus) was 0.021 for sub-population 1 and 0.047 for sub-population 2 of cotton germplasm with a mean value of 0.072. The mean value of I (Shannon’s Information Index) was 0.908 and 0.909 for sub-populations 1 and 2 of cotton germplasm, respectively. Gene diversity (*h*) value was 0.544 for sub-population 1 while it was 0.538 for sub-population 2 with an average of 0.541 in the whole cotton germplasm. Unbiased diversity (uh) value was slightly higher for each sub-population as compared to gene diversity (h), *viz.* 0.562 and 0.546 for sub-population 1 and sub-population 2, respectively, with an average of 0.554 (Supplementary Table 4 and Supplementary Figure 2).

### Linkage Disequilibrium

**FIGURE 4:**
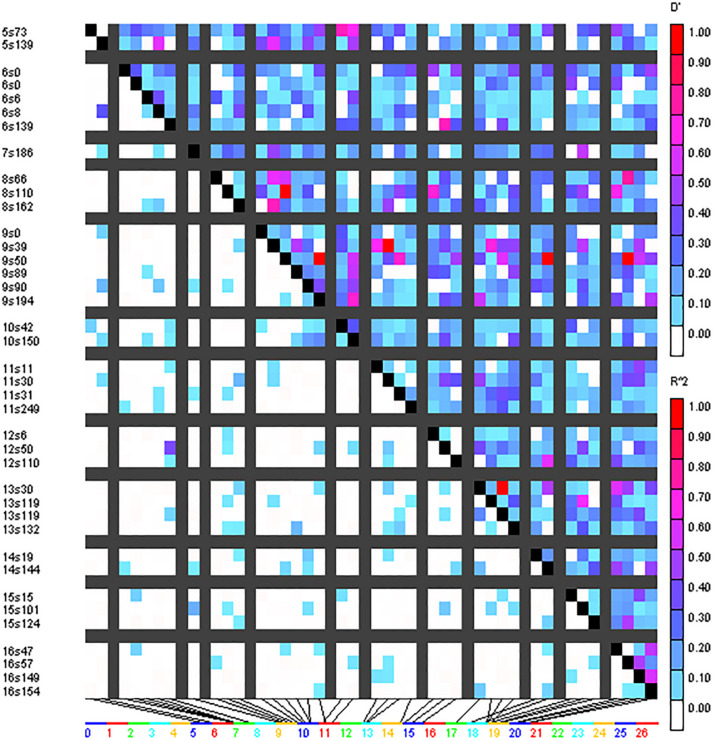
Triangle heat plot showing pairwise value of *D*’ and *r*^2^ for different locus pair over different chromosomes of cotton in the present germplasm panel.

A total of 293 SSR marker-based alleles were used to calculate the extent of LD resulting in pairwise LD detection in 4656 locus pairs for the cotton genotype panel. Out of 4656 locus pairs, a total of 514 SSR marker pairs (11.04%) showed significant LD at the threshold (i.e., *r*^2^ ≥ 0.05) (Supplementary Table 5). Out of 514 SSR marker pairs, 17 were collinear, i.e., markers on the same chromosome, and 497 were inter-chromosomal. At considerably higher levels, i.e., *r*^2^ ≥ 0.1, significant LD was obtained for 151 marker pairs (3.24%). Out of these 151 marker pairs, 7 marker pairs were collinear while the remaining 144 were inter-chromosomal. LD blocks were observed as demonstrated by triangle plots for pairwise LD between SSRs (Figure 4). Sizes of intra-chromosomal LD blocks were also calculated; at *r*^2^ ≥ 0.1 in 26 chromosomes, the longest LD block (211 cM) was observed on chromosome 23 between the marker pairs BNL4053 and BNL1672. Out of seven significant collinear LD blocks, four were on chromosome 23, and the remaining three were on chromosome 9, chromosome 13, and chromosome 19.

### Association Mapping

In order to have significant MTAs, a number of approaches, *viz.* MLMM, CMLM, and MLM, implemented in Genomic Association and Prediction Integrated Tool (i.e., GAPIT) were used. MLMM was considered as a basic approach and it was further supported by CMLM and MLM models. Environment-wise, different significant MTAs were identified. Quantile–quantile (QQ) plots between observed and expected *p* values of association by MLMM model revealing the fitting of the model for all the six environments are given in Supplementary Figure S3.

**FIGURE 5:**
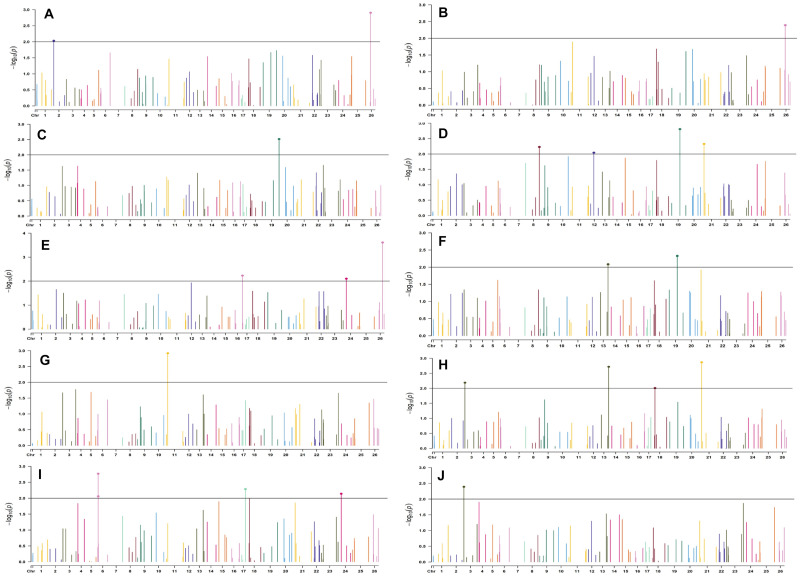
Manhattan plots for the early sown environment of 2018 for various traits under study by the MLMM model. Significant marker above threshold {at –log10 (*p*) > 2} is highlighted with bold dot head on the marker’s line. **(A)** Days to first flower, **(B)** plant height, **(C)** number of monopods, **(D)** number of bolls per plant, **(E)** boll weight, **(F)** seed cotton yield per plant, **(G)** ginning out turn, **(H)** lint yield, **(I)** seed index, and **(J)** lint index.

**FIGURE 6:**
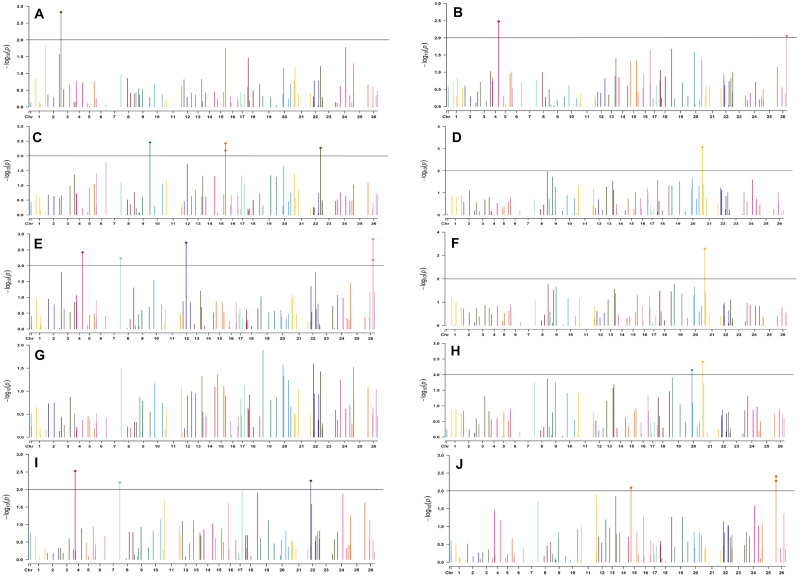
Manhattan plots for the early sown environment of 2019 for various traits under study by the MLMM model. Significant marker above threshold {at –log10 (*p*) > 2} is highlighted with bold dot head on the marker’s line. **(A)** Days to first flower, **(B)** plant height, **(C)** number of monopods, **(D)** number of bolls per plant, **(E)** boll weight, **(F)** seed cotton yield per plant, **(G)** ginning out turn, **(H)** lint yield, **(I)** seed index, and **(J)** lint index.

In the early sown-2018 environment, a total of 32 markers were involved in 56 significant MTAs (since a single marker can be associated with multiple traits) at *p* < 0.01. Out of 56 significant MTAs identified, a maximum of 12 MTAs were associated with SI while a minimum of 2 MTAs were associated with NM (Supplementary Table S6). However, in the early sown-2019 environment, 24 markers were involved in 32 significant MTAs at *p* < 0.01. Out of 32 MTAs identified, a maximum of five associated with PH, NM, SI, and BW, while for NB and GOT, one marker each was associated, i.e., BNL3279-120 and BNL2961-241, respectively (Supplementary Table S7). Graphical representations of significant associations using Manhattan plot for early sown environments for the year 2018 and 2019 are given in Figures 5, 6, respectively.

In the early sown-2018 environment, three markers located on chromosome 19, *viz.* BNL2961 (with BW and SI), CGR5732 (with NB, GOT, and SCY/P), and BNL3452 (with NB, SCY/P, and LY); two markers on chromosome 3, *viz*. BNL3441 (with NB, LY, and LI) and BNL3279 (NB and LY); two markers on chromosome 13, *viz*. BNL1551 (BW and SI) and BNL3479 (with SCY and LY); one marker on chromosome 20, *viz.* JESPR 220 (with GOT, SCY/P, and LY); one marker on chromosome 6, *viz*. BNL4108 (with SI and LI); one marker on chromosome 18, *viz*. BNL1721 (with NB and GOT); and one marker on chromosome 12, *viz*. BNL3423 (with DF, PH, GOT, and LI) were significantly associated with two or more traits. However, for early sown-2019, BNL3976 (with BW and SI) on chromosome 7, BNL3279 (NB, SCY/P, and LY) on chromosome 3, BNL4028 (NM, SCY/P, and LY) located on chromosome 9, BNL2572 (PH and BW) on chromosome 4, BNL686 (DF and NM) on chromosome 15, and CGR5452 (PH and BW) on chromosome 12 were significantly associated to more than one trait. Seven markers, i.e., BNL1605, BNL3279, BNL448, BNL3280, BNL296, BNL 3441, and BNL4108, were common in both years for the early sown environments.

**FIGURE 7:**
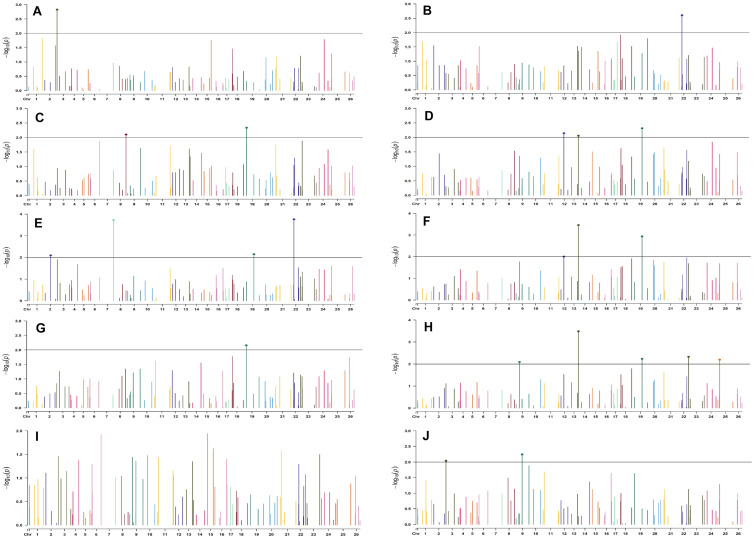
Manhattan plots for the normal sown environment of 2018 for various traits under study by the MLMM model. Significant marker above threshold {at –log10 (*p*) > 2} is highlighted with bold dot head on the marker’s line. **(A)** Days to first flower, **(B)** plant height, **(C)** number of monopods, **(D)** number of bolls per plant, **(E)** boll weight, **(F)** seed cotton yield per plant, **(G)** ginning out turn, **(H)** lint yield, **(I)** seed index, and **(J)** lint index.

**FIGURE 8:**
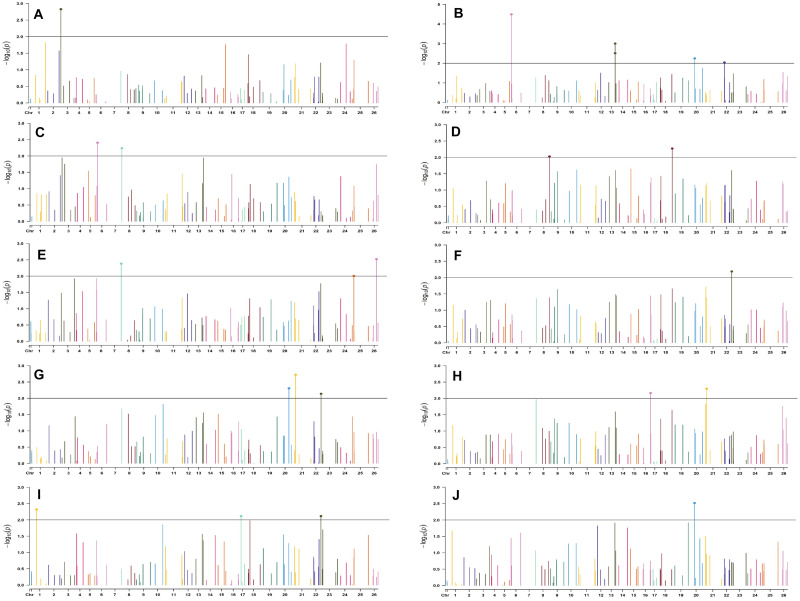
Manhattan plots for the normal sown environment of 2019 for various traits under study by the MLMM model. Significant marker above threshold {at –log10 (*p*) > 2} is highlighted with bold dot head on the marker’s line. **(A)** Days to first flower, **(B)** plant height, **(C)** number of monopods, **(D)** number of bolls per plant, **(E)** boll weight, **(F)** seed cotton yield per plant, **(G)** ginning out turn, **(H)** lint yield, **(I)** seed index, and **(J)** lint index.

For normal sown environments of both years (2018 and 2019), 24 and 30 markers were involved in 46 and 49 significant MTAs, respectively, at *p* < 0.01 (Supplementary Tables 8, 9). Out of 46 identified significant MTAs in the normal sown environment of 2018, a maximum of 11 were associated with SCY/P while a single marker was significantly associated with PH; however, no significant MTA was found to be associated with BW at *p* < 0.01 in this environment. For normal sown-2019, out of 49 significant MTAs, a maximum of nine were associated with NM and a minimum of two each were associated with DF, NB, and LI. Graphical representation of significant MTAs using Manhattan plot for normal sown environments for the year 2018 and 2019 are given in Figures 7, 8, respectively.

For the normal sown-2018 environment, two markers located on chromosome 19, *viz.* BNL3452 (with NM and GOT) and CGR5732 (with NB, SI, SCY/P, and LY); two markers on chromosome 12, *viz.* CGR5452 (with NB and SCY/P) and BNL3423 (with GOT and LI); two on chromosome 18, *viz.* BNL1721 (with NB, SCY/P, and LY) and BNL2544 (SI and LI); one marker on chromosome 10, i.e., CGR5565 (with GOT, SI, and LI); BNL3479 on chromosome 13 (NB and SCY/P); one marker on chromosome 20, *viz.* JESPR 220 (with SCY/P and LY); one marker on chromosome 3, *viz*. BNL3441 (with DF and LI); one marker on chromosome 15, *viz*. BNL686 (with DF and LY); and one on chromosome 6, *viz*. NAU2355 (with SCY and LY) were significantly associated with two or more traits. However, in the normal sown-2019 environment, BNL3976 located on chromosome 7 (with BW and NM), BNL686 (with DF, GOT, SI, and SCY/P) on chromosome 15, BNL1066 (with GOT and LY) on chromosome 3, BNL3994 (with PH and LI) on chromosome 20, BNL3085 (with NM and SCY) on chromosome 1, and BNL1551 (with NM and LY) on chromosome 13 were significantly associated with more than one trait. Nine markers, i.e., BNL3479, BNL3976, BNL3257, BNL2921, JESPR220, CGR5732, BNL3441, BNL686, and BNL3482, were common in the normal sown environment for both years.

**FIGURE 9:**
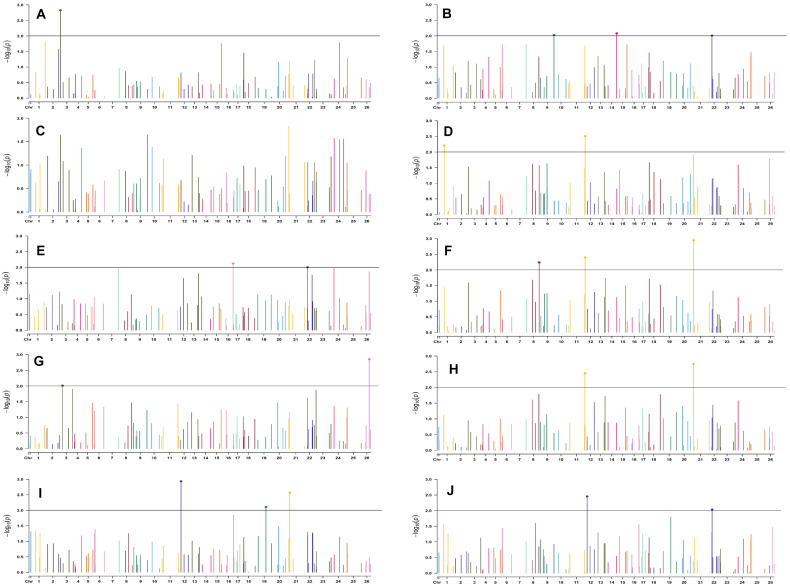
Manhattan plots for the late sown environment of 2018 for various traits under study by the MLMM model. Significant marker above threshold {at –log10 (*p*) > 2} is highlighted with bold dot head on the marker’s line. **(A)** Days to first flower, **(B)** plant height, **(C)** number of monopods, **(D)** number of bolls per plant, **(E)** boll weight, **(F)** seed cotton yield per plant, **(G)** ginning out turn, **(H)** lint yield, **(I)** seed index, and **(J)** lint index.

**FIGURE 10:**
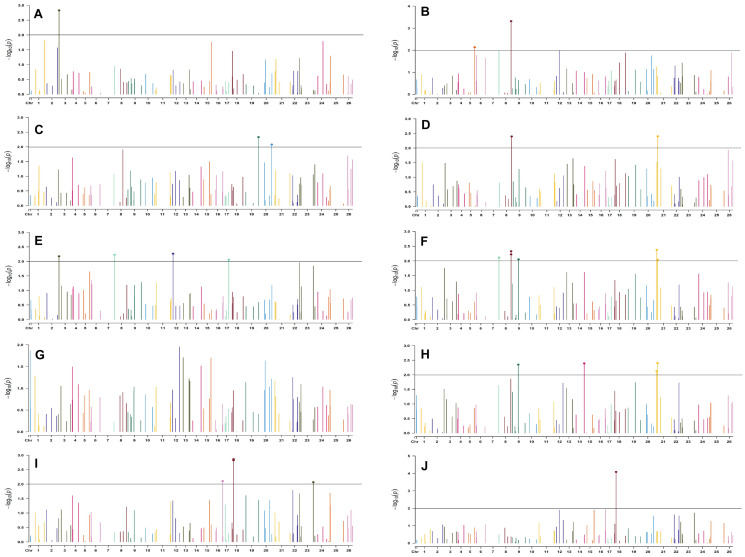
Manhattan plots for the late sown environment of 2019 for various traits under study by the MLMM model. Significant marker above threshold {at –log10 (*p*) > 2} is highlighted with bold dot head on the marker’s line. **(A)** Days to first flower, **(B)** plant height, **(C)** number of monopods, **(D)** number of bolls per plant, **(E)** boll weight, **(F)** seed cotton yield per plant, **(G)** ginning out turn, **(H)** lint yield, **(I)** seed index, and **(J)** lint index.

For late sown-2018 and 2019 environments, 34 and 26 markers were involved in 53 and 48 significant MTAs, respectively, at *p* < 0.01 (Supplementary Tables 10, 11). Out of 53 significant MTAs in the late sown environment of 2018, a maximum of 12 significant MTAs were identified for SI, and a minimum of 2 MTAs were identified for DF and LY. In the late sown-2019 environment, out of 48 significant MTAs, a maximum of 8 significant MTAs were identified for BW and SCY/P but a minimum of 2 for DF. Graphical representations of significant MTAs using Manhattan plot for late sown environments for the year 2018 and 2019 are given in Figures 9, 10, respectively.

In the late sown-2018 environment, markers BNL3261 (with GOT, SI, and LI) and CGR5452 (with PH and NM) on chromosome 12, BNL3452 (NB and SI) mapped on chromosome 19, BNL1681 (NB, SI, SCY/P, and LY) on chromosome 1, BNL4108 (NM, SI, and LI) on chromosome 6, BNL448 (with BW and LI) on chromosome 20, BNL3279 (with SCY and LY) on chromosome 3, CGR5161(with NM and LI) on chromosome 8, BNL3099 (PH and SCY) on chromosome 9, and BNL2590 (SI and LI) on chromosome 15 were significantly associated with two or more traits. However, in the late sown-2019 environment, BNL1066 (with NB, SCY/P, and LI), BNL3441 (with DF, PH, and BW), and BNL3279 (with SCY/P and LI) on chromosome 3; BNL2847 (with SCY and LY) and BNL3099 (with GOT and LY) on chromosome 9; BNL686 (with DF, SI, and LI) on chromosome 15; BNL4035 (with SI and LI) on chromosome 18; BNL3482 (with NM and LI) on chromosome 20; BNL3257 (with PH, NB, and SCY/P) on chromosome 8; BNL3368 (with NB, SCY/P, and LY) on chromosome 26; and BNL3976 (associated to BW, SI, and SCY/P) located on chromosome 7 were significantly associated with more than one trait. Fifteen markers, *viz.* BN448, BNL4035, BNL3261, BNL3998, BNL1066, BNL3257, BNL1551, BNL2590, BNL3279, BNL3441, BNL3502, BNL3976, BNL4035, BNL686, and BNL3099, were found to be common in the late sown environment of both the years.

To find out MTAs with high confidence, we have identified some markers comparing the results of all six environments and MTAs, which were common to at least two environments and were reported as promising MTAs. Twenty-five markers were found to be involved in 38 significant MTAs at *p* < 0.01, which were common to at least two environments (Table 3). Nine markers, *viz.* CGR5732 (NB, SI, and SCY/P) on chromosome 19, BNL3976 (BW and SI) on chromosome 7, BNL3257 (NB and SCY/P) on chromosome 8, BNL4108 (SI and LI) on chromosome 6, BNL3479 (SCY and LY) on chromosome 13, JESPR220 (SCY and LY) on chromosome 20, BNL1721 (NB and LY) on chromosome 18, BNL3423 (GOT and LI) on chromosome 12, and BNL3279 (NB, SCY, and LY) on chromosome 3 were significantly associated with more than one trait at *p* < 0.01.

**TABLE 3:** List of significant marker–trait associations which were common to at least two environments.

SSR	Trait	Chromosome	Position (cM)	PVE (R2)	References
BNL3441-210	DF	3	0	_	Yu et al., 2013; Abdullaev et al., 2017
BNL686-140	DF	15	1	0.081–0.084	
BNL1053-210	PH	1	243	0.121–0.155	
BNL1605-100	BW	8	90	_	Li et al., 2017
BNL1551-170	BW	13	149	_	
BNL827-168	BW	10	26.3	0.201	Shen et al., 2006; Abdurakhmonov et al., 2009; Li et al., 2016
BNL2572-260	BW	4	92.7	0.125	Sun et al., 2012
BNL3976-165	BW	7	186	_	
CGR5732-175	NB	19	130	_	
BNL3279-120	NB	3	22.9	_	
BNL3257-230	NB	8	162	_	
CGR5452-160	NB	12	50	_	
BNL3452-170	NB	19	18.9	0.123–0.232	Abdullaev et al., 2017; Dong C. et al., 2018
BNL3452-200	NB	19	18.9	0.123–0.232	Abdullaev et al., 2017; Dong C. et al., 2018
BNL1721-200	NB	18	31	0.098–0.136	
BNL3998-180	NM	19	210	_	
BNL448-222	NM	20	17.9	0.151–0.173	He et al., 2007; Kantartzi and Stewart, 2008; Zhang et al., 2013
BNL3423-220	GOT	12	45.5	_	
CGR5565-146	GOT	10	150	0.098–0.155	Zhang et al., 2013
BNL3099-160	GOT	9	144	0.092–0.135	Wu et al., 2009; Sun et al., 2012
CGR5534-141	SI	2	90	0.100–0.109	Li et al., 2017; Mei et al., 2017
BNL4108-180	SI	6	139	0.096–0.143	Abdurakhmonov et al., 2009; Li et al., 2016
CGR5732-175	SI	19	130	0.093	
BNL2590-190	SI	15	211	0.125–0.138	Abdullaev et al., 2017
BNL3976-130	SI	7	186	0.120–0.204	
CGR5732-175	SCY/P	19	130	_	
BNL3479-240	SCY/P	13	132	_	Abdullaev et al., 2017
JESPR220-171	SCY/P	20	29.2	0.104–0.111	Zhang et al., 2013
BNL3279-120	SCY/P	3	22.9	_	
BNL3257-210	SCY/P	8	162	_	
BNL3279-120	LY	3	22.9	_	
BNL3479-240	LY	13	132	_	Abdullaev et al., 2017
BNL1721-200	LY	18	31	0.119	
JESPR220-161	LY	20	29.2	0.127	
JESPR220-171	LY	20	29.2	0.11–0.127	
BNL1066-150	LY	3	35.6	_	
BNL4108-180	LI	6	139	0.122–0.154	Abdurakhmonov et al., 2009; Li et al., 2016
BNL3423-240	LI	12	45.5	0.122–0.172	

Out of 38 high confidence markers, 22 MTAs were identified as novel. A number of markers significantly associated with lint yield and its contributing traits have shown linkage or pleiotropic effect in the present study (Table 4).

**TABLE 4:** List of markers having linkage and pleiotropic effect (i.e., significantly associated to two or more traits).

Marker	DF	PH	BW	NB	NM	GOT	SI	LI	SCY/P	LY
BNL1551			✓		✓		✓			✓
CGR5732				✓				✓	✓	✓
BNL3452				✓					✓	
BNL3279				✓				✓	✓	✓
BNL3479				✓					✓	✓
BNL1721				✓					✓	✓
BNL3976			✓	✓	✓				✓	
BNL686	✓					✓		✓	✓	
BNL1681				✓			✓			
BNL4108					✓		✓	✓		
BNL1066				✓		✓			✓	✓
BNL3257		✓		✓					✓	
BNL3368				✓					✓	✓

*DF, days to flower; PH, plant height; BW, boll weight; NB, number of boll per plant; NM, number of monopods per plant; GOT, ginning out turn; SI, seed index; SCY/P, seed cotton yield per plant; LY, lint yield; LI, lint index.*

Significant MTAs linked to various traits were further analyzed by identifying the genes and their functions. Moreover, locations of the associated genes were identified using the reference genome of *G. hirsutum* available at COTTONGEN resource (see footnote 1). It showed that out of 25 markers identified (Table 3), 10 markers could be properly annotated with underlying candidate genes and their functions (Table 5).

**TABLE 5:** List of annotated markers associated with candidate genes and their possible function.

Markers	Gene	GO term	Description
BNL3279	Gh_A11G2619	GO0006006|	Glyceraldehyde/Erythrose phosphate dehydrogenase family
		GO:0016620|	
		GO:0050661|	
		GO:0051287|	
		GO:0055114	
BNL3423	Gh_A12G2146		Lin-54 family
BNL3479	Gh_D13G1262	GO0006486|	Glycosyltransferase family 10 (fucosyltransferase)
		GO:0008417|	
		GO:0016020	
BNL3998	Gh_D05G1102	GO0004672|	Serine-threonine/tyrosine-protein kinase catalytic domain
		GO:0005524|	
		GO:0006468	
BNL827	Gh_D06G0084		Protein of unknown function (DUF616)
BNL1551	Gh_D11G2627	GO:0005634|	XRN 5′-3′ exonuclease N-terminus (Zinc finger CCHC-type profile)
		GO:0006139	
BNL1605	Gohir.D12G051500	GO0005515	BRCT domain
CGR5534	Gh_A02G0114	GO0003677|	No apical meristem (NAM) protein
		GO:0006355	
CGR5565	Gh_A10G0216	GO0005515|	A domain family that is part of the cupin metalloenzyme superfamily
		GO:0008270	
BNL448	Gh_A05G3315	GO0007034	Snf7 family

## Discussion

The selection of a highly diverse germplasm or genotype panel is the key requirement for the success of any association mapping study. Statistical analysis (ANOVA) of seed cotton yield and its contributing traits in the present study showed that significant variation was present between genotypes and the date of sowing had also affected the performance of genotypes. Except for NM, all other traits had varied significantly in different sowing environments. It further suggests that a suitable genotype panel has been selected for the study. Buttar et al. (2010) also reported that NM, sympods, and plant height could not vary significantly in different sowing environments. Zhao et al. (2012) and Jamro et al. (2017) also reported similar type of results. It showed that the performance and productivity of upland cotton can be higher when the crop is sown on or before the normal sowing time (before May 10). Variation for means and range of LY and its contributing traits in the same sowing environments of 2 years was at par as compared with different sowing environments of the same year. A similar pattern of variation has been reported in previous studies for upland cotton (Bozbek et al., 2006; Khan et al., 2017). Estimation of phenotypic correlation among the recorded traits showed that seed cotton yield has a significant and highly positive correlation with BW and NB irrespective of the environment. So, the selection for these two traits in yield improvement program will increase the lint yield. Similar patterns of correlation were reported in previous studies by Ul-Allah et al. (2017), Kumar et al. (2019), and Mahdi and Emam (2020). Results of ANOVA, the performance of genotypes in all six environments, and correlation among the quantitative traits gave the assurance to proceed further with the present panel of genotypes for the association mapping.

### Molecular Diversity

The PIC value for 97 polymorphic SSR markers ranged from 0.126 (NAU1093) to 0.693 (BNL1721), with an average value of 0.485, which was higher as reported by Ersoz et al. (2007), Kalivas et al. (2011), Wang et al. (2011), Zhao et al. (2014), Qin et al. (2015), and Nie et al. (2016). Range (0.126–0.693) and average (0.485) of PIC showed that the panel of markers used in the present study was sufficient to explore the genetic variation among the genotypes of the present study. PIC value was in agreement with the results of Tu et al. (2014) (0.46), Wang et al. (2016) (0.47), and Dong C. G. et al. (2018) (0.48). All 97 polymorphic markers were distributed over 26 chromosomes with an average of 3.73 markers per chromosome. Markers were equally distributed on both of the genomes (A and D) of tetraploid *G. hirsutum*. A total of 47 (i.e., 48%) and 50 (i.e., 52%) SSR markers were located over all the 13 chromosomes of A- and D-genome each, respectively. A total of 293 different alleles were amplified by the present panel of SSRs, ranging from 2 to 5 alleles per marker with an average value of 3.020 alleles per marker, which is lower than as reported by Abdullaev et al. (2017) and Baytar et al. (2017). The results for the number of alleles for markers depend on the panel of germplasm, but these are also highly affected even by the methods used for screening of results like PAGE, AGE, MAZE, micro-capillary, etc. Results of the present study were in accordance with Fang et al. (2013); Jia et al. (2014), and Zhao et al. (2014). This diversity is highly useful for plant breeders while selecting the genotypes for varietal or hybrid development. Genotypes that were highly diverse at the molecular level too will give a more heterotic effect in their F_1_ hybrids. Results are in accordance with previous reports as genotypes or lines from different origins showed higher genetic distance as compared with genotypes of origin (Fang et al., 2013; Mei et al., 2013; Nie et al., 2016; Kaur et al., 2017). Though the panel of genotypes in the current study is limited to Indian origin only, still it has enough diversity to proceed further.

AMOVA showed that higher variation is present among the genotypes as compared to between the groups. A similar fashion of variation was reported in previous studies as, for example, higher variation within the genotypes of a group was reported by Abdurakhmonov et al. (2008) (96.7%), Zhao et al. (2015) (86%), Nie et al. (2016) (96%), Tyagi et al. (2014) (65.8%), Jena et al. (2012) (70.8%), and Kaur et al. (2017) (66.7%). AMOVA and other genetic diversity analyses showed that genotypes used in the present study are diverse and could be used in the cotton development program.

### Population Structure and LD

By using the results obtained from STRUCTURE analysis, the maximum likelihood value of Δ*K* was recorded for *K* = 2 followed by *K* = 9 and *K* = 3. So, the genotypes were grouped into two sub-groups. The reason for a smaller number of significant clusters might be the same geographical origin of most of the genotypes in the present study and close ancestral history. Mei et al. (2013); Cai et al. (2014), and Song et al. (2019) reported the highest likelihood score at Δ*K* = 2 and obtained clusters with large germplasms grouping into two clusters, while Jena et al. (2012), Tyagi et al. (2014), Badigannavar and Myers (2015), Kaur et al. (2017), and Seyoum et al. (2018) reported likelihood score at *K* = 3 or more and clustered the genotype panels accordingly.

Significant linkage was obtained for 151 marker pairs (3.24%) at *r*^2^ ≥ 0.1 and for 514 markers pairs (11.04%) at *r*^2^ ≥ 0.05, which were similar to results as reported in previous studies by Cai et al. (2014), Li et al. (2016), Iqbal and Rahman (2017). The collinear LD was concentrated on chromosomes 9, 13, and 23. The presence of larger LD blocks in triangle plots for pairwise LD might be due to selection pressure on the genotypes or varieties in breeding programs for specific desirable traits. In north Indian regions, the extent of cross-pollination in cotton is much lesser, which possibly may have reduced the frequency of recombination.

### Association Mapping

Association between seed cotton yield traits and SSR markers was estimated by three different models, which are MLMM, CMLM, and MLM, so that the model that best fits the results of association mapping could be identified. The strategy of using two or more different models for detection of favorable MTAs in cotton has been practiced previously in several studies (Badigannavar and Myers, 2015; Wang et al., 2016; Abdullaev et al., 2017; Baytar et al., 2017; Sethi et al., 2017), and comparative results of those models were considered. Confirmation of MTAs with different models over the different environmental conditions gave more confidence in the results and reduces the chances of false association. The results of LD for 97 SSRs were motivation to proceed to association mapping as, previously, Cai et al. (2014) conducted the association mapping using 99 polymorphic SSR markers in *G. hirsutum* and Abdullaev et al. (2017) conducted the same association mapping analysis in *G. barbadense* using 108 polymorphic SSR markers. Recently, several other association studies were also conducted using a low number of markers and genotypes in the cotton. Badigannavar and Myers (2015) used 64 polymorphic primer combinations to detect association for seed quality traits in 75 upland cotton germplasm, Iqbal and Rahman (2017) used 95 polymorphic SSRs to detect the association for fiber quality traits in upland cotton, and Ali et al. (2020) used 22 markers to detect association for yield contributing traits in 28 genotypes of upland cotton.

Thirty-eight significant MTAs for lint yield and its contributing traits (which were common to at least two environments) were considered promising MTAs. Out of these 38 MTAs, 16 were in agreement with previous studies, either for the same or for different traits of cotton. A total of 22 MTAs identified in the present study were found to be novel. Fifteen markers had also shown linkage or pleiotropic effect in this study. Among 38 promising markers, CGR5732 showed significant association with number of bolls per plant, seed index, and seed cotton yield per plant. BNL3257 was reported to be significantly associated with number of bolls per plant and seed cotton yield per plant. BNL3976 was found to be significantly associated with boll weight and seed index. BNL3279 showed significant association with number of bolls per plant, seed cotton yield per plant, and lint yield. BNL3423 showed significant association with ginning out turn and lint index and JESPR220 with seed cotton yield per plant and lint yield. Out of these four markers, BNL3279 had been reported to be associated with fiber quality traits in previous studies by Abdullaev et al. (2017) and Dong C. G. et al. (2018). Markers associated with fiber quality in previous studies were found to be associated with seed cotton yield contributing traits in the present study. It had shown that SSR markers used for seed cotton yield contributing traits could also be helping in fiber quality trait improvement simultaneously. In earlier studies (Iqbal and Rahman, 2017; Zhang et al., 2020), it has been reported that SSR markers associated with boll weight, number of bolls per plant, and ginning out turn were also significantly associated with fiber quality traits.

A number of markers significantly associated with lint yield and its contributing traits have shown linkage and pleiotropic effect in the present study. These markers could be used in other studies too for the screening of lint- and fiber-related traits. SSR markers have shown similar linkage and pleiotropic effect in previous studies of Abdullaev et al. (2017), Iqbal and Rahman (2017), and Zhang et al. (2020). Use of these markers showing linkage and pleiotropic effect in cotton will be very helpful in the screening of progenies in crop improvement programs specific to yield and other yield attributing traits.

### Annotation

Marker BNL3279 associated with traits like NB and SCY/P was found to be linked to gene Gh_A11G2619, which produces Glyceraldehyde/Erythrose phosphate dehydrogenase family (Zhang et al., 2015) and helps in fruit development in plants. BNL3479 associated with NB and SCY/P was found to be linked to gene Gh_D13G1262, which produces Glycosyltransferase family 10 (fucosyltransferase) and helps in cell wall biosynthesis (Hansen et al., 2012). BNL3998 associated with traits like NM and GOT was found to be linked to gene Gh_D05G1102, which synthesizes serine-threonine/tyrosine-protein kinase catalytic domain. Marker BNL1551 associated with BW, NM, and SI was found to be linked to gene Gh_D11G262 synthesizing XRN 5′-3′ exonuclease N-terminus (Zinc finger CCHC-type profile), which is a multifunction protein (Kastenmayer and Green, 2000). Marker CGR5565 associated to GOT and SI was found to be linked to gene Gh_A10G0216, which produces a domain family that is part of the cupin metalloenzyme superfamily and which has been reported to be involved in the growth and development of plants (Dunwell et al., 2004). Marker BNL3423 associated with GOT was found to be associated with Gene Gh_A12G2146, which produces proteins of Lin-54 family required for cell cycle progress (Schmit et al., 2009). Results of annotation showed that markers found in MTAs and linked to functional genes need fine mapping and further research for more authenticity in the results and further use of these markers in the crop improvement programs.

## Conclusion

LD-based association mapping was conducted in 96 genotypes of upland cotton with 97 polymorphic markers. Extensive phenotyping of studied genotypes in different sown environments for two consecutive years showed that significant variation was available among the genotypes for lint yield and its attributing traits. Based on molecular variation detected by 97 polymorphic markers, all genotypes were significantly divided into two clusters. Association mapping done by the three most appropriate methods detected the number of markers associated with different traits of the study. Out of 38 promising MTAs identified in the present study, 22 SSR alleles were considered as novel while the remaining marker alleles were in agreement with previous studies. Annotation of the markers with functional genes showed that the outcome of the present study will help in the genetic improvement of lint yield in crop improvement programs.

## Data Availability Statement

The raw data supporting the conclusions of this article will be made available by the authors, without undue reservation.

## Author Contributions

PK conducted the research and wrote the manuscript. SN and RS supervised the research and reviewed the manuscript. NB and DM analyzed the data and reviewed the manuscript. VS, Sagar, and RC assisted in wet lab work and gave their suggestions in the manuscript. All the authors contributed to the article and approved the submitted version.

## Conflict of Interest

The authors declare that the research was conducted in the absence of any commercial or financial relationships that could be construed as a potential conflict of interest.

## Footnotes

1 https://www.cottongen.org/find/markers
