# Supplementary material for: Identification of Novel Marker–Trait Associations for Lint Yield Contributing Traits in Upland Cotton (Gossypium hirsutum L.) Using SSRs
Source: Front Plant Sci. 2021 May 26;12:653270. doi: 10.3389/fpls.2021.653270 (PMC8187916; doi:10.3389/fpls.2021.653270)
Supplement: Supplementary file 1 [file Data_Sheet_1.docx]

**Table S1 List of selected diverse 96 genotypes used in the present study**

| **Sr. No.** | **Genotype No.** | **Pedigree** |
| --- | --- | --- |
| 1 | CBMH-1 | H 974 x J 34 P2-3-1-1-1-1 |
| 2 | CBMH-4 | H1117 x H 1226 P1-1-1-1-1 |
| 3 | CBMH-7 | H1226 x PIL 8 P2-2-2-1-3-2-1 |
| 4 | CBMH-9 | H 1226 x RS 875 P5-5-4-1-1-1-1 |
| 5 | CBMH-10 | JK 1050 BG II P2-1-1-2-1-1 |
| 6 | CBMH-11 | Pancham P2-1-1-1-1-1 |
| 7 | CBMH-12 | Khakhi Kapas P1-1-3-1-1 |
| 8 | CBMH-16 | F 2228 x H1117 P1-2-1-1-1-1 |
| 9 | CBMH-18 | H 1508 P1 |
| 10 | CBMH-19 | Bio 6488-2 BG II P2-1-1-2 |
| 11 | CBMH-24 | GM 19-1-2-1 |
| 12 | CBMH-27 | GM 27-2-1-1 |
| 13 | CBMH-28 | GM 34-2-2-2 |
| 14 | CBMH-29 | GM 33-1-1-1 |
| 15 | CBMH-32 | H 1226 X RS 875 P2-3-1-1-1-1 |
| 16 | CBMH-35 | Coker P2-1-1-1-1 |
| 17 | CBMH-36 | H974 x RS 875 P1-2-2-1-1 |
| 18 | CBMH-37 | H1117 x H 1226 P 3-3-1-1-1-1 |
| 19 | CBMH-39 | H 1226 x RS 875 P3-8-3-1-1-1 |
| 20 | CBMH-41 | H 1226 X H1256 P1-5-1-1 |
| 21 | CBMH-42 | H 1490 P1-2 |
| 22 | CBMH-46 | H 1098I X H 1442 P1-1-1-2-1 |
| 23 | CBMH-47 | H 1477 P2—2 |
| 24 | CBMH-49 | LH 2108 X H 1098 P5-1-1-2-1 |
| 25 | CBMH-50 | H 1470 X H 1316 P3-1-1-1-1 |
| 26 | CBMH-52 | H 1470 X H 1316 P5 |
| 27 | CBMH-53 | H 1481 X H 1353P1 |
| 28 | CBMH-54 | H 1489 P1—1 |
| 29 | CBMH-55 | H 1481 X H 1465 P2 |
| 30 | CBMH-56 | H 1480 X H 1353 P1 |
| 31 | CBMH-58 | GTHH-217 P1 |
| 32 | CBMH-59 | FHH 261P2 |
| 33 | CBMH-60 | H 1487 P1-2-1 |
| 34 | CBMH-61 | RCH 791P3-1-2-1-1-1 |
| 35 | CBMH-62 | Ankur 3224 P1-1-1-2-1 |
| 36 | CBMH-63 | H1475 x H 1300P1-1-1-1-1 |
| 37 | CBMH-64 | PCH 402 Bt P1-1-2-1-1 |
| 38 | CBMH-65 | PCH 402 Bt P1-2-3-1-1 |
| 39 | CBMH-66 | PCH 402 Bt P2-1-1-1-1 |
| 40 | CBMH-68 | NCS 9030 BGII P2-4-2-1-1 |
| 41 | CBMH-69 | NCS 1916 Bt P1-4-1-1-1 |
| 42 | CBMH-70 | Deltapine 66 |
| 43 | CBMH-71 | Fergusan |
| 44 | CBMH-72 | Sharda |
| 45 | CBMH-74 | Unknown |
| 46 | CBMH-75 | P22 |
| 47 | CBMH-76 | P25 |
| 48 | CBMH-77 | PRT 35 |
| 49 | CBMH-78 | J 2 P 7 |
| 50 | CBMH-79 | 321 |
| 51 | CBMH-80 | Mex 685-3 |
| 52 | CBMH-81 | HS90-80 |
| 53 | CBMH-83 | Bio-100-P3-1-1-2-1 |
| 54 | CBMH-84 | Bio-100-P3-5-1-1-1 |
| 55 | CBMH-85 | Bio-100-P3-10-1-1-1 |
| 56 | CBMH-87 | Bio-100-P12-2-1-1-1 |
| 57 | CBMH-89 | Bio-100-P12-5-1-1-1 |
| 58 | CBMH-92 | GCH-3-5 |
| 59 | CBMH-93 | GCH-3-1 |
| 60 | CBMH-94 | GCH-3-3 |
| 61 | CBMH-95 | GCH-3-2 |
| 62 | CBMH-96 | GCH-3-5 |
| 63 | CBMH-97 | H 1487 P1-4 |
| 64 | CBMH-99 | H 1481 P2-2 |
| 65 | CBMH-102 | H1117 P2-P1-1 |
| 66 | CBMH-103 | Bio-100-P56-1-3-1-1 |
| 67 | CBMH-104 | Bio-100-P56-2-5-2-1 |
| 68 | CBMH-105 | Bio-100-P56-3-6-1-1 |
| 69 | CBMH-106 | Bio-100-P56-4-7-1-1 |
| 70 | CBMH-107 | Bio-100-P57-3 |
| 71 | CBMH-108 | Bio-100-P57-4 |
| 72 | CBMH-109 | Bio-100-P57-9 |
| 73 | CBMH-110 | Bio-100-P57-3-4 |
| 74 | CBMH-113 | Bio-100-P57-8-5 |
| 75 | CBMH-114 | Bio-100-P3-1-2-1-1 |
| 76 | CBMH-115 | Bio-100-P7-3 |
| 77 | CBMH-118 | Bio-100-P19-1 |
| 78 | CBMH-121 | Bio-100-P32-4 |
| 79 | CBMH-123 | Bio-100-P41-1 |
| 80 | CBMH-124 | Bio-100-P41-5 |
| 81 | CBMH-125 | Bio-100-P45-6 |
| 82 | CBMH-126 | Bio-100-P49-1 |
| 83 | CBMH-127 | Bio-100-P49-2 |
| 84 | CBMH-128 | GCH-3-1-4 |
| 85 | CBMH-131 | H 1481 P1-1-1-1 |
| 86 | CBMH-132 | H 1487 P1-4-1-1 |
| 87 | CBMH-133 | GM 37-3-P1-2-4 |
| 88 | CBMH-134 | GM 4-2 P5-1-124 |
| 89 | CBMH-135 | H 1481 P2-1-5 |
| 90 | CBMH-136 | H 1481 P2-5-1 |
| 91 | CBMH-137 | H1117 P2-P1-1-3 |
| 92 | CBMH-140 | Bio-100-P56-6-7 |
| 93 | CBMH-142 | Bio-100-P57-4-7 |
| 94 | CBMH-143 | GM 4(General Mean)-1-P1-1-4 |
| 95 | CBMH-144 | GM 4(General Mean)-1-P1-2-4 |
| 96 | CBMH-145 | GM 4-2-P2-2-3 |

**Table S2: A brief description of SSR primers used during the present investigation**

| **Marker name** | **Chromosome** | **Forward sequence** | **Reverse sequence** |
| --- | --- | --- | --- |
| BNL0827 | 10 | AAGCTCCACGTGCTCAAGTT | CTCATGTTGTCGGTGGTGTT |
| BNL0830 | 2 | TTCCGGGTTTTCAATAAACG | GTTAATACTTTTTTTCTTTTGTGTGTG |
| BNL1030 | 9,23 | TTTGGAGCCATTTACATGCA | AAACCACTTCTGCATCTGGA |
| BNL1053 | 21 | AGGGTCTGTCATGGTTGGAG | CATGCATGCGTACGTGTGTA |
| BNL1061 | 10 | GCTTGTCATCTCCATTGCTG | TAGCCCGGTTCATGTTCTTC |
| BNL1064 | 6 | TTTGCGGGTAATCCTATTGC | TGTCTATGGGACATTTCGCA |
| BNL1066 | 7 | ACATTTCCACCCAAGTCCAA | ACTCTATGCCGCCTCTCGTA |
| BNL1122 | 1 | TCGATAACGGCTATAGTAATCTCTC | CAACAAATAAGCAGCCAAGAAA |
| BNL1231 | - | TAATAAAAGGGAAAGGAAAGAGTT | TATGGCTCTAGAATATTCCCTCG |
| BNL1317 | 6 | AAAAATCAGCCAAATTGGGA | CGTCAACAATTGTCCCAAGA |
| BNL1395 | 1 | AAGCAGCCAAGAAATACCGA | TCGATAACGGCTACAGTAATCT |
| BNL1414 | 9,23 | AAAAACCCCTTTCCATCCAT | GGGTGTCCTTCCCAAAAATT |
| BNL1421 | 13 | TGAAGATTTGGAGGCAATTG | GAAATCAAGCCTCAATTCGG |
| BNL1434 | 5 | AAATTCAAGAATCAAAAAACAACA | TTATGCCAAAGTATATGGAGTAACG |
| BNL1440 | 6, 25 | CCGAAATATACTTGTCATCTAAACG | CCCCCGGACTAATTTTTCAA |
| BNL1495 | 13 | TGAAGATTTGGAGGCAATTG | ATAAATGGCATCAGCCCAAA |
| BNL1521 | 4 | TGAAGAAAGAAAAAGAGAAAGGG | CTCACCACGTGGCACTTATG |
| BNL1551 | - | CGCAAGCCACCTGTAAAAC | TCGAATTTTCTCTCTCTCTCTCTCT |
| BNL1605 | 5,8 | TTGGGGAGAATGAAGGAGAG | TATCTACTGCCAGCGCTCCT |
| BNL1606 | 3 | CATGTAGGATGAGAGAGAGAGAGAGA | GGGGCTTTACGACATACCTG |
| BNL1672 | 6 | TGGATTTGTCCCTCTGTGTG | AACCAACTTTTCCAACACCG |
| BNL1681 | 7 | GTGTGTGGGTGTGCATGTTT | TGGGGAGACTTTATCACGCT |
| BNL1693 | 1,15 | CCCTTGGGAATAGCAGGTG | CATGTGTCTCCGTGTGTGTGTG |
| BNL1694 | 1 | CGTTTGTTTTCGTGTAACAGG | TGGTGGATTCACATCCAAAG |
| BNL1705 | 7 | GCCAATTTAGTATAGGAAGCAAGT | CATGTATTATTTTCACCCCTCTCT |
| BNL1707 | 9,12 | TCCTAGGCTGAGTGAGGGCT | AATGACGTCGTTTTATGCCC |
| BNL1721 | 18 | TGTCGGAATCTTAAGACCGG | GCGCAGATCCTCTTACCAAA |
| BNL2495 | 8 | ACCGCCATTACTGGACAAAG | AATGGAATTTGAACCCATGC |
| BNL2499 | 4 | TTTCTTCTTTCAAAACACACACG | GAAACTCATGGGGAACCAAA |
| BNL2544 | 13 | GCCGAAACTAAAACGTCCAA | TCCTTACTCACTAAGCAGCCG |
| BNL2570 | 11 | TTCTACAAAAAAAGAAAAAATGGG | AAATACGGATGGGACCAACC |
| BNL2572 | 4 | GTCCTATTACTAAAATTGTTAATTTAGCC | CGATGTTAAATCAATCAGGTCA |
| BNL2590 | 6 | GAAAAACCAAAAAGGAAAATCG | CTCCCTCTCTCTAACCGGCT |
| BNL2651 | 5 | CGAGTGTCTCCAAAACATCG | AGATGATGAGGGGGAGGTTT |
| BNL2847 | 9 | AACAATGCCGGAAGCTAGTG | CTTTTCTTGCTGCCTCTGCT |
| BNL2921 | 1 | CGAGAGATTTTAAAGGGAAACA | GGGAGTGGTCTGATGGAAAA |
| BNL2961 | D3 | TCGAAAGGGTGTTTCTTCTT | GGGGATGCTTGTCACATCTT |
| BNL3031 | 6 | AGGCTGACCCTTTAAGGAGC | AACCAACTTTTCCAACACCG |
| BNL3065 | 16 | CAAACGGGAGACCAAAAAAA | CGAACTGGCGAGTTAGTGCT |
| BNL3085 | 1,15 | TGGACATCCTTCTGGAAACC | TGTGGAGTCATCAATATGTTGC |
| BNL3089 | 4 | TCGAACTTAACAAAAGAGAGAGAGA | AAATCCGGATTCAGCAATACTT |
| BNL3099 | 14,20 | GCCCATGTTCAAATCAATCA | CCCCGACCTGAATCTAACCT |
| BNL3257 | 8 | CAATCTGGGATCAAAAAAACC | GGTGAAACATAGCGTGTTGC |
| BNL3259 | 5,10 | TTTTGAAATTCCAGCGAAGG | GTCAATACCTGCTTCTCCACG |
| BNL3261 | 12 | AAACGGAAACGAAGAAGGGT | CCCAAACCTGTCTCACCAAC |
| BNL3279 | 7 | CATGTCCAATGGATGTGTCA | GGGCCACTTAAAGGCATTCT |
| BNL3280 | 13 | GCAGAACTGCCACTTGTTTG | AGAAAATGGGTTGTGCTTGG |
| BNL3359 | 6 | TTGTTGTTGGGAATGATGGA | TGACCCTTCACCGACTTTCT |
| BNL3368 | 8 | TAGAACACGGGGGAAAACAG | CATTGAAACCCCATCAAAGG |
| BNL3398 | 3 | TATACCAATTTATTTCAAAACACACA | TTCATCGTAGCACAATCACACAT |
| BNL3408 | 3 | ATCCAAACCATTGCACCACT | GTGTACGTTGAGAAGTCATCTGC |
| BNL3423 | 8 | CTGTAGCTCGTGGCTGACTG | ATCTTCAGCGGTGGGTGTAG |
| BNL3436 | 10 | AACATAGCCTACCATTGCCG | TTGTTTGCCAAATTTGAAGC |
| BNL3441 | 3 | CGTCATAAACCGTGCTTGTG | GGCCACTTTAAGGCTGTCAC |
| BNL3452 | 9 | TGTAACTGAGCAGCCGTACG | GCCAAAGCAGAGTGAGATCC |
| BNL3479 | 13 | AGTGGGTTGGACTTTCATGC | CACGGGCTTTTTTTTTTTCA |
| BNL3482 | 20,26 | ATTTGCCCCAGGTTTTTTTT | GCAACACCTTTTCCTCCCTA |
| BNL3502 | 5 | AATTTCTAAGATAACACACAAACACA | TACAATCAAATAGCAGTTTAGAGTATCG |
| BNL3563 | 10 | AAGCATAAACTTGACACAAGCC | AATGGGCAAGAAAAGGGAAC |
| BNL3590 | 3 | TCTTCCCTCTCTTTCTCTTTCG | ACACGGAAGACCAACCAAGT |
| BNL3594 | 1,10 | AGGGATTTTGATTGTTGTGC | TGAATTCAAAACAAATGTTAGCC |
| BNL3649 | 7 | GCAAAAACGAGTTGACCCAT | CCTGGTTTTCAAGCCTGTTC |
| BNL3816 | 8 | GTTAGCCACGTGTTAGTTCTATG | ATCGATCACTTGCTGGTTCC |
| BNL3835 | 4,12 | ATTTGGTCATTTTGGTGTATGG | TGCATAATAAAGGGCATTTACA |
| BNL3867 | 12,26 | TAATTGAGTTGTTTTCTTACTTGCC | TGCCAATTTAGCAATCACCA |
| BNL3902 | 15 | GAGTTTGGGGGCTGTGTATG | GGGGTGCTTATGTCAGACGT |
| BNL3904 | 8 | ATGCATTAATGAGTCGATAGGC | GCACAAAGAAAACAAACTGTACG |
| BNL3948 | 11 | GTAATGTTCAACACTTTGCTATTCC | GTTGGTTGGGTGAGCAGAAT |
| BNL3976 | 5,7 | AAACACCCCCTGCTACTTCA | AGAACAGAAGGAGAGACACCG |
| BNL3994 | 4,22 | TTGAGGGCATCCAAATCCAT | CCTCCACCATACACGTGCTA |
| BNL3998 | 9 | CGGCGAGAAGTTGAAAGATC | TGTGCAAAAGTGGGTGGTAA |
| BNL4028 | 9 | CTCGAATCACTGTTCCGACA | TTGTTGCGGATCATTGGATA |
| BNL4034 | 3 | GAGGAAGGATGGCTAACTGGTATAA | GTCAAAACACATATCACACACACAC |
| BNL4035 | 20 | TGCATCTGCATTTGGGATTA | TAGCCAACCGTTACACACCA |
| BNL4053 | 6 | TGAAGGCTTTGAAGCAAACA | AAGCAAGCACCAAGTTAGCC |
| BNL4060 | 5 | TCGATGGAAGTGTAAGAATTCG | ACGCCGAAAAGGGAGTTACT |
| BNL409 | 13 | AGAAGTCGGACGTGGAAAGA | GCCGTTTTCCTCAGTGATGT |
| BNL4108 | A6 | TCCACCATTCCCGTAAATGT | TGGCCAAGTCATTAGGCTTT |
| BNL448 | AD20/AD22 | GCAGCTTGCTTTTCTGCTTC | ACGCAAGCTTGGTCAATACC |
| BNL686 | 6 | ATTTTTCCCTTGGTGGTCCT | ACATGATAGAAATATAAACCAAACACG |
| BNL786 | 15 | CTTTCCACGTGTAATTTGTTGATA | GATCTTAACTCTTGCTCTCTCTCTCTC |
| BNL827 | 10 | AAGCTCCACGTGCTCAAGTT | CTCATGTTGTCGGTGGTGTT |
| BNL946 | 11 | GCTGTTGCTCCACATCTCCT | GGGCAAACAGATAGGCAGAA |
| CGR5031 | 9 | GCAAGCAGCAGTGATAATGG | AACATGCAAGCAAACAAGCA |
| CGR5161 | 8 | CTTTGGAGTTAGGAACATTAGC | GGTGATAATAACTACTCTGACGACGA |
| CGR5193 | 8 | GGCATCAGGTGCCCTCTTA | AGCAAGTCCGGCACAATC |
| CGR5282 | 2 | AGGCTCCTTATGACGTGGG | ACCTCCTTCCCTCACACCTT |
| CGR5452 | 8 | ATCCCGGGAAAGTCACAGTT | TATCGGCAACAATGGTGTTT |
| CGR5534 | 2 | TCAAGTGCAGCAAGGATGTC | TGAACACTGGCGTTCTGAAG |
| CGR5565 | 20 | GCCATTAACCCATTAGGCAA | GCCATTGGAGCTTATAAGGATG |
| CGR5576 | 17 | CGGTTCAACCCGACTGTTT | GAGGAAAGAAAGGAAGAGAGGG |
| CGR5578 | 21 | ACCACCCGATACCCAAAGAT | GTGCCGCCACTGGTAAACT |
| CGR5707 | 6 | AAACCCGATATCCTTAGCCTTT | GGAAAGGAGGAAGAGGAGGA |
| CGR5732 | 5 | GCCAAGGTTCATTCCTGAAA | TATAGGGCTCATCAGGGTGG |
| CGR5871 | 5 | TTACCGGGTCTGGGATATTG | ATGCAGCTTCGGAATCTCTG |
| CGR6012 | 12 | ATGTGCCTTGTCCTCTTCCA | GGGATGTGAGGGCATCATAG |
| CGR6022 | 20 | TGTTTGGCATAAACCCGAAG | TTCTCTATAACCTCTACCCGCCTA |
| CGR6078 | 1 | CATGCAAGAAAGCTGCTCAA | TAGGCATGTGTCTCCGTGTG |
| CGR6103 | 4 | CAAAGGATGGGACACAGGTAA | TGCATTAGATACCGAAATGAGC |
| CGR6110 | 11 | CGCAGGTGACTCAAACTCTG | GGTAAGACTATGTAGATGCTTACATGC |
| CGR6254 | 12,26 | TTCGTTCGTCGCTTTCTTCT | TTGAAGATCCAACTTTGCCC |
| CGR6356 | 1 | ACCCACCACAGATCTGCAA | CAGATCACATCCTACTTCGTCAA |
| CGR6807 | 2 | TGCTGAAACGAAACCTCGTA | AAGTGACAAATCCACTGCCC |
| CGR6880 | 8 | CGGCTGTATTTGTTTGGTGC | GGGATGTGAGGGCATCATAG |
| JESPR110 | 2,17,23,17 | GGCGAAGAGCTACCTGTGAATGGC | CCAATGGGGACTCTACATGTGGC |
| JESPR152 | 15 | GATGCACCAGATCCTTTTATTAG | GGTACATCGGAATCACAGTG |
| JESPR153 | 13,18, 20 | GATTACCTTCATAGGCCACTG | GAAAACATGAGCATCCTGTG |
| JESPR197 | 05, 05 | CAATACCTGGAACATAGACAAATG | CTTGAGGCTTGCAAAAAATG |
| JESPR204 | 5,13, 19, 18, | CTCCAGGTTCAATGGTCTG | GCCATGTTGGACAAGTAGTC |
| JESPR208 | D09, 23, F2 | CGCAACCAAACATATACTTCACAC | CCCTTTCCATCCATAGAACG |
| JESPR220 | D04, 22 | CGAGGAAGAAATGAGGTTGG | CTAAGAACCAACATGTGAGACC |
| JESPR232 | 08, F2-A02 | CAGACCACGCTATTTTTGCC | CGTTGTATTATTTCCAGTGCTCG |
| JESPR270 | l, 13,26, 15 | ACGCAACTCGCATATAAACAC | GTAGCTTAGAATTTGAATGGC |
| JESPR274 | 9, 23, 26, | GCCCACTCTTTCTTCAACAC | TGATGTCATGTGCCTTGC |
| JESPR291 | 24,8, 24/D8 | CATTCCCCACTTTGCTCTTAC | CATGTTTCTTTGCCCATC |
| NAU 1093 | 6 | AGCCTGCATGTTCTATCTCC | TTCCCTTTTAATTGGGACCT |
| NAU 1103 | D11-2, 21, 11 | GGAGCCAGAAGTTGAGAAAA | TTCGGCTTCTGCTTTTACTT |
| NAU 2238 | 8 | TTTTTCATGGCTGAACTTTG | ATTTCCATTGCTCGCTTTAC |
| NAU0934 | A05 | TGCTTTCGTATCCTTTTTCC | ATTAGAGAAGCCAGGGAGGT |
| NAU0943 | 12, 12/A12 | ATCTGTTCAATTTCTCGTCA | CAGTTGTTGGTTGATCTGGA |
| NAU0998 | A03 | CTCTCTCTCACACACGCACT | CTGGCCAAGTGACACAATTA |
| NAU1025 | 23, 23/D9 | GAAGGACCGTTTTGTCTAA | TTCAGAATAGGTGGGCCTAA |
| NAU1037 | D08, 8/A8 | CACCTTCACCTAACCATCAA | GAAGAATTGCGAGAAGAGGA |
| NAU1042 | 5,19, D08 | CATGCAAATCCATGCTAGAG | GGTTTCTTTGGTGGTGAAAC |
| NAU1048 | 7 | GGCCATATTATTGCAGAACC | ACAGCCTTGAGTTGAGCTTT |
| NAU1070 | A03 | GGCCATATTATTGCAGAACC | ACAGCCTTGAGTTGAGCTTT |
| NAU1085 | 7 | AGTCGCCCCTTCTCTAATTT | TGTAAACCGAACTCGTTGTG |
| NAU1102 | D05 | ATCTCTCTGTCTCCCCCTTC | GCATATCTGGCGGGTATAAT |
| NAU1103 | D11-2, 21, 11 | GGAGCCAGAAGTTGAGAAAA | TTCGGCTTCTGCTTTTACTT |
| NAU1141 | 13, 18 | CCCCTCTCTCTGTTTCTCAA | AAGGGGTTTGAAGGGTTATC |
| NAU1156 | 5-2, 19 | ACACTCTCTCAGCTGGAACC | GGTCTCCCTCTAGCTTGTTG |
| NAU1167 | 3, 17, A03 | CTGACTTGGACCGAGAACTT | AAGAGCCCTGGACAATGATA |
| NAU1190 | A03 | CCATGTCCGTATCCATGTTA | TAAGGCAAGATAGGGTCAGG |
| NAU1211 | 13-Feb | CCTTCATTTCTCTCCTCCAA | GATACGAGGTCGTTTTGGTC |
| NAU1218 | A06 | TGTGATGAAGAACCCTCTCA | CACTCAACCCAATGAAACAA |
| NAU1230 | A05 | CATGCAAATCCATGCTAGAG | TCAAAAGGTTCTTTGGTGGT |
| NAU1233 | A11, 10/A10 | TTCGGGAAAGTTAGAGGAGA | TCCTCAGAGCTCGGAATAGT |
| NAU1366 | 12, 21/IgD02 | CATGAAGCTTTTCCCACTTT | CAGCTTATCCACCCCTAATG |
| NAU1369 | D08 | TGGCAGAGATGAATGTAAGC | GGTAACGGATGGAAAATCAC |
| NAU2078 | 7, 16-1 | GTAGGGCTTTCCGATACTGA | ATCCTGCAATAAGGCGTTC |
| NAU2083 | 1, 01/A1 | AGAAGAGGTTGACGGTGAAG | TGAGTGAAGAACCTGCACAT |
| NAU2162 | 22, 04, 22/D4 | ACACAAAAACCCAAAGGAAA | CACGAGTGTCCTTGCTACAG |
| NAU2165 | 15, 15/D1 | TAAATTTTGAGATGGCAGCA | CAAGGTGAAGGCAAAGAGAT |
| NAU2169 | D08 | AGCAAATCCAATCACTTGGT | AAAAGTAGCGGGATGAGATG |
| NAU2190 | 14, 14/D2 | CAACCAACATATTCCAAACA | TTATTTCGGCCTTGTTTTTC |
| NAU2200 | 9, 09/A09-2 | TCTCAAAATTTACACAGCATCC | GCCTCGTATATCTCCATTGC |
| NAU2251 | 12, 26, 12/A12 | TTCTCCAGTAACCAACAAAGG | AAAATATCATCCCCGTCAAA |
| NAU2257 | A11 | GACTACCACCCACACTTCAAC | TTGGTGTGTGGCAAATGTAT |
| NAU2265 | 2 | CAATCACATTGATGCCAACT | CGGTTAAGCTTCCAGACATT |
| NAU2306 | 24, 24/D8 | TCGGGTACCTGTAAGAGAGC | TGTTGCCGAATAACAATCAC |
| NAU2317 | 10/A10 | GACTCCAGCCTTCACACAT | TGGAAGAGTATAACGGCAGA |
| NAU2336 | 14, 14/D2 | TGGAAAAGGAAGAGGAGAGA | CCCTGAAGTTGTCAAGCTCT |
| NAU2354 | 9, 09/A9 | AATATCTCCGTCGCCAATTA | GAAACTTCCTCCTCCTTTCC |
| NAU2355 | A1 | ACAAACAAAACGCCTTCTTC | AACACAAAAACGGTTCCAGT |
| NAU2419 | 01, 01/A1 | TCAAAACATTGGCATTGAAC | CACCAAATTCAGGTTCACAA |
| NAU2439 | 24, 24/IgA02 | GGCAACACAACAAAGGACTA | CACTTGCTGCAGTTTACCAC |
| NAU2443 | 18, 18/D13 | CGTTGAGAAGGAAAGCCTAA | AGCCTGCTTCATGTTCTTTT |
| NAU2477 | 4, 22 | CACGAGTGTCCTTGCTACAG | ACACAAAAACCCAAAGGAAA |
| NAU2503 | 19, 19/D5 | GATCGAAATCAAAGGGCTTA | CGTTGGATGAAGTTGATGAT |
| NAU2508 | 10/A10 | TGGAGGAGGGTGTAACATCT | GGCATTCAAGGAGATGAGTT |
| NAU2581 | 06-3 | GACTTGGGTGGTGGATAAAC | GTAGCTTCACGGTGTCCTCT |
| NAU2641 | 25, 25/D6 | TCTTTTGAGGGTCACCATTT | AACCCAGTTTTTGTTTTCCA |
| NAU2666 | 09, 09/A09-2 | TCTCAAAATTTACACAGCATCC | TGATCTGGTTTTGGATTTGA |
| NAU2672 | 12, 04, 12/ A12 | GGTTCCGAAACTTTGTTGTT | TGATGGTGGCGATGATAATA |
| NAU2679 | 06, 25 | TGGCTGAACTTTGCAATTTA | AAGCAAGGGAGGTAATCCTT |
| NAU2687 | 25 | CTGAGACTGTCCATGTCCAA | ATCTGGGTTTTCCCTTTTTC |
| NAU2691 | 17, 17/D3 | TCACATCTTGCAAGCTCATT | AGTTAAAACCGGGCTGAGAT |
| NAU2697 | 18, 18/D13 | ATTTCCCATGGTCATAGCAG | GAAAGGAGTCGGAAATGAGA |

**Table S3 Major allele frequency, gene diversity and PIC value of 97 polymorphic SSR markers**

| S. No. | Marker | Major Allele Frequency | Gene Diversity | PIC |
| --- | --- | --- | --- | --- |
| 1 | BNL827 | 0.432 | 0.698 | 0.647 |
| 2 | BNL1030 | 0.823 | 0.291 | 0.249 |
| 3 | BNL1053 | 0.602 | 0.554 | 0.49 |
| 4 | BNL1061 | 0.75 | 0.375 | 0.305 |
| 5 | BNL1066 | 0.692 | 0.47 | 0.419 |
| 6 | NAU1093 | 0.927 | 0.135 | 0.126 |
| 7 | NAU1141 | 0.813 | 0.305 | 0.258 |
| 8 | NAU1156 | 0.865 | 0.234 | 0.207 |
| 9 | NAU1167 | 0.781 | 0.342 | 0.283 |
| 10 | NAU1190 | 0.379 | 0.661 | 0.587 |
| 11 | NAU1233 | 0.604 | 0.478 | 0.364 |
| 12 | NAU1369 | 0.75 | 0.375 | 0.305 |
| 13 | BNL1672 | 0.573 | 0.489 | 0.37 |
| 14 | NAU2083 | 0.698 | 0.422 | 0.333 |
| 15 | NAU2162 | 0.771 | 0.353 | 0.291 |
| 16 | NAU2355 | 0.76 | 0.364 | 0.298 |
| 17 | BNL3031 | 0.677 | 0.437 | 0.342 |
| 18 | BNL3089 | 0.854 | 0.249 | 0.218 |
| 19 | BNL3649 | 0.563 | 0.492 | 0.371 |
| 20 | JESPR220 | 0.677 | 0.477 | 0.417 |
| 21 | JESPR274 | 0.885 | 0.203 | 0.182 |
| 22 | BNL1122 | 0.823 | 0.301 | 0.27 |
| 23 | BNL1395 | 0.792 | 0.33 | 0.275 |
| 24 | BNL1421 | 0.44 | 0.647 | 0.573 |
| 25 | BNL1434 | 0.813 | 0.305 | 0.258 |
| 26 | BNL1440 | 0.802 | 0.317 | 0.267 |
| 27 | BNL1495 | 0.453 | 0.645 | 0.573 |
| 28 | BNL1605 | 0.564 | 0.582 | 0.515 |
| 29 | BNL1606 | 0.79 | 0.332 | 0.277 |
| 30 | BNL1694 | 0.844 | 0.264 | 0.229 |
| 31 | BNL1705 | 0.771 | 0.353 | 0.291 |
| 32 | BNL1707 | 0.688 | 0.429 | 0.337 |
| 33 | BNL2495 | 0.854 | 0.249 | 0.218 |
| 34 | BNL3976 | 0.415 | 0.658 | 0.593 |
| 35 | BNL3835 | 0.615 | 0.548 | 0.488 |
| 36 | BNL3398 | 0.406 | 0.646 | 0.571 |
| 37 | BNL3482 | 0.448 | 0.604 | 0.52 |
| 38 | BNL786 | 0.531 | 0.607 | 0.539 |
| 39 | BNL686 | 0.446 | 0.711 | 0.67 |
| 40 | BNL409 | 0.39 | 0.702 | 0.649 |
| 41 | BNL3994 | 0.448 | 0.649 | 0.578 |
| 42 | BNL3576 | 0.442 | 0.62 | 0.539 |
| 43 | BNL3368 | 0.396 | 0.661 | 0.587 |
| 44 | BNL2499 | 0.5 | 0.659 | 0.608 |
| 45 | BNL2544 | 0.552 | 0.602 | 0.542 |
| 46 | BNL2570 | 0.406 | 0.656 | 0.582 |
| 47 | BNL3441 | 0.385 | 0.665 | 0.594 |
| 48 | BNL3590 | 0.542 | 0.594 | 0.523 |
| 49 | BNL3479 | 0.458 | 0.643 | 0.571 |
| 50 | BNL2847 | 0.49 | 0.621 | 0.547 |
| 51 | BNL4053 | 0.563 | 0.588 | 0.523 |
| 52 | BNL3259 | 0.49 | 0.63 | 0.558 |
| 53 | BNL3359 | 0.448 | 0.644 | 0.571 |
| 54 | BNL3257 | 0.479 | 0.629 | 0.556 |
| 55 | BNL3065 | 0.646 | 0.519 | 0.464 |
| 56 | BNL3998 | 0.406 | 0.658 | 0.584 |
| 57 | BNL3436 | 0.625 | 0.542 | 0.488 |
| 58 | BNL4034 | 0.448 | 0.643 | 0.569 |
| 59 | BNL4035 | 0.531 | 0.607 | 0.539 |
| 60 | BNL3085 | 0.646 | 0.457 | 0.353 |
| 61 | BNL2590 | 0.479 | 0.627 | 0.553 |
| 62 | BNL3902 | 0.5 | 0.62 | 0.547 |
| 63 | BNL1064 | 0.51 | 0.5 | 0.375 |
| 64 | BNL3280 | 0.406 | 0.654 | 0.58 |
| 65 | BNL3423 | 0.448 | 0.692 | 0.642 |
| 66 | BNL3452 | 0.385 | 0.717 | 0.666 |
| 67 | BNL3563 | 0.313 | 0.739 | 0.691 |
| 68 | BNL3948 | 0.625 | 0.469 | 0.359 |
| 69 | BNL448 | 0.479 | 0.678 | 0.63 |
| 70 | CGR5031 | 0.396 | 0.66 | 0.589 |
| 71 | BNL2961 | 0.344 | 0.724 | 0.673 |
| 72 | BNL1640 | 0.406 | 0.646 | 0.571 |
| 73 | BNL3816 | 0.438 | 0.643 | 0.568 |
| 74 | BNL3261 | 0.458 | 0.687 | 0.637 |
| 75 | BNL946 | 0.458 | 0.64 | 0.566 |
| 76 | BNL2651 | 0.594 | 0.482 | 0.366 |
| 77 | BNL3099 | 0.333 | 0.739 | 0.692 |
| 78 | BNL1551 | 0.615 | 0.547 | 0.487 |
| 79 | BNL4028 | 0.406 | 0.657 | 0.583 |
| 80 | CGR5452 | 0.427 | 0.649 | 0.575 |
| 81 | BNL2572 | 0.313 | 0.736 | 0.688 |
| 82 | CGR5578 | 0.396 | 0.685 | 0.626 |
| 83 | CGR5161 | 0.583 | 0.567 | 0.501 |
| 84 | CGR5193 | 0.667 | 0.492 | 0.435 |
| 85 | BNL3279 | 0.438 | 0.633 | 0.559 |
| 86 | BNL2921 | 0.406 | 0.661 | 0.594 |
| 87 | CGR5534 | 0.573 | 0.489 | 0.37 |
| 88 | CGR5565 | 0.448 | 0.693 | 0.643 |
| 89 | CGR5282 | 0.292 | 0.735 | 0.685 |
| 90 | CGR5576 | 0.438 | 0.699 | 0.65 |
| 91 | CGR5707 | 0.896 | 0.187 | 0.169 |
| 92 | CGR5732 | 0.313 | 0.736 | 0.687 |
| 93 | BNL1693 | 0.385 | 0.704 | 0.651 |
| 94 | BNL1721 | 0.333 | 0.74 | 0.693 |
| 95 | BNL3502 | 0.469 | 0.638 | 0.565 |
| 96 | BNL4108 | 0.385 | 0.653 | 0.578 |
| 97 | BNL1681 | 0.396 | 0.657 | 0.583 |

**Table S4 Genetic diversity and mean allelic pattern across sub-populations of cotton germplasm**

| **Population** | **Sub-Population 1** | | **Sub-Population 2** | | **Total Mean** |
| --- | --- | --- | --- | --- | --- |
|  | **Mean** | **SE** | **Mean** | **SE** |  |
| **Na** | 2.887 | 0.076 | 2.990 | 0.085 | 2.938 |
| **Na Freq. >= 5%** | 2.845 | 0.075 | 2.897 | 0.075 | 2.871 |
| **Ne** | 2.406 | 0.070 | 2.403 | 0.074 | 2.404 |
| **I** | 0.908 | 0.030 | 0.909 | 0.031 | 0.908 |
| **NP** | 0.021 | 0.015 | 0.124 | 0.047 | 0.072 |
| **No. L Comm Alleles (<=25%)** | 0.000 | 0.000 | 0.000 | 0.000 | 0.000 |
| **No. L Comm Alleles (<=50%)** | 0.000 | 0.000 | 0.000 | 0.000 | 0.000 |
| **H** | 0.544 | 0.015 | 0.538 | 0.016 | 0.541 |
| **Uh** | 0.562 | 0.016 | 0.546 | 0.017 | 0.554 |

**Na** = No. of Different Alleles per locus, **Ne** = No. of Effective Alleles per locus, **NP**= No. Private Alleles per locus, **I** = Shannon's Information Index, **h** = Gene Diversity, **uh** = Unbiased Diversity

**Table S5 Extent of linkage disequilibrium (LD) between SSR loci at whole genome level in 96 cotton genotypes**

| **Parameters** | ***r*² ≥ 0.1** | ***r*² ≥ 0.05** |
| --- | --- | --- |
| Samples size | 96 | 96 |
| Collinear LD % (LD/non-LD) | 4.93(7/142) | 11.97(17/142) |
| Inter-chromosomal LD % (LD/non-LD) | 3.20(144/4514) | 11.01(497/4514) |
| Total LD% (LD/non-LD) | 3.24(151/4656) | 11.04(514/4656) |

**Table S6 Significant marker-trait associations in early sown 2018 environment using three different methods i.e. MLMM, CMLM and MLM**

| **Trait** | **SSR** | **Chromosome** | **Position** | ***p* value** | | | ***r*^2^ (PVE in %)** | | |
| --- | --- | --- | --- | --- | --- | --- | --- | --- | --- |
|  |  |  |  | **MLMM** | **CMLM** | **MLM** | **MLMM** | **CMLM** | **MLM** |
| DF | BNL3423-220 | NaN | 45.5 | 0.0012** |  |  |  |  |  |
| DF | CGR5282-156 | 2 | 10 | 0.0093* |  |  |  |  |  |
| DF | BNL3902-180 | 15 | 14.6 |  | 0.0031** | 0.0030** |  | 11.48 | 10.61 |
| PH | BNL2921-132 | 1 | 46 |  | 0.0070* | 0.0070* |  | 13.86 | 13.86 |
| PH | BNL3259-220 | 3 | 184 |  | 0.0074* | 0.0074* |  | 13.75 | 13.75 |
| PH | BNL3423-220 | NaN | 45.5 | 0.0040** |  |  |  |  |  |
| NM | BNL3998-180 | 19 | 210 | 0.0031** |  |  |  |  |  |
| NM | BNL448-222 | 20 | 17.9 |  | 0.0015** | 0.0019** |  | 16.63 | 15.14 |
| NB | CGR5732-175^ | 19 | 130 | 0.0016** |  |  |  |  |  |
| NB | BNL3279-120 | 3 | 22.9 | 0.0047** |  |  |  |  |  |
| NB | BNL3257-230 | 8 | 162 | 0.0059* |  |  |  |  |  |
| NB | CGR5452-160 | 12 | 50 | 0.0090* |  |  |  |  |  |
| NB | BNL3452-170^ | 19 | 18.9 |  | 0.0024** | 0.0024** |  | 12.27 | 12.27 |
| NB | BNL3452-200 | 19 | 18.9 |  | 0.0044** | 0.0044** |  | 10.96 | 10.96 |
| NB | BNL1721-200 | 18 | 31 |  | 0.0077* | 0.0077* |  | 9.77 | 9.77 |
| NB | BNL3441-200 | 3 | 0 |  |  | 0.0022** |  |  | 12.43 |
| BW | BNL1605-100 | 8 | 90 | 0.0002*** |  |  |  |  |  |
| BW | BNL1551-170^ | 13 | 149 | 0.0059* |  |  |  |  |  |
| BW | BNL2961-231 | 19 | 18.1 | 0.0079* |  |  |  |  |  |
| GOT | BNL3948-101 | 1 | 30.5 | 0.0012** |  |  |  |  |  |
| GOT | BNL3948-110 | 1 | 30.5 | 0.0012** |  |  |  |  |  |
| GOT | CGR5732-164 | 19 | 130 |  | 0.0026** |  |  | 11.28 |  |
| GOT | BNL3280-240 | 18 | 26 |  | 0.0035** | 0.0016** |  | 10.62 | 12.13 |
| GOT | BNL3423-220 | 12 | 45.5 |  | 0.0052* |  |  | 9.77 |  |
| GOT | CGR5565-146 | 10 | 150 |  | 0.0068* | 0.0060* |  | 9.21 | 9.28 |
| GOT | BNL3099-160 | 9 | 144 |  | 0.0097* |  |  | 8.45 |  |
| GOT | JESPR220-161 | 20 | 29.2 |  |  | 0.0082* |  |  | 8.61 |
| SI | BNL3359-210 | 6 | 6 | 0.0017** |  |  |  |  |  |
| SI | BNL3590-170 | 2 | 61.5 | 0.0052* |  |  |  |  |  |
| SI | BNL2961-221 | 19 | 18.1 | 0.0072* |  |  |  |  |  |
| SI | BNL3359-220 | 6 | 6 | 0.0087* |  |  |  |  |  |
| SI | NAU1093-174 | 10 | 4.3 |  | 0.0023** | 0.0029** |  | 13.58 | 12.72 |
| SI | NAU1093-180 | 10 | 4.3 |  | 0.0023** | 0.0029** |  | 13.58 | 12.72 |
| SI | BNL1551-170^ | 13 | 149 |  | 0.0035** | 0.0026** |  | 12.73 | 12.93 |
| SI | BNL3368-155 | 26 | 111 |  | 0.0074* | 0.0083* |  | 11.14 | 10.51 |
| SI | BNL2570-240 | 20 | 141 |  | 0.0082* | 0.0071* |  | 10.93 | 10.84 |
| SI | CGR5534-150 | 2 | 90 |  | 0.0083* |  |  | 10.90 |  |
| SI | CGR5534-141 | 2 | 90 |  | 0.0083* |  |  | 10.90 |  |
| SI | BNL4108-180 | 6 | 139 |  | 0.0094* | 0.0054* |  | 10.65 | 11.39 |
| SCY/P | CGR5732-175^ | 19 | 130 | 0.0047** |  |  |  |  |  |
| SCY/P | BNL3479-240 | 13 | 132 | 0.0083* |  |  |  |  |  |
| SCY/P | BNL946-239 | 20 | 77 |  | 0.0054* | 0.0054* |  | 10.55 | 10.55 |
| SCY/P | JESPR220-171 | 20 | 29.2 |  | 0.0059* | 0.0059* |  | 10.35 | 10.35 |
| SCY/P | BNL3452-170^ | 19 | 18.9 |  | 0.0088* | 0.0088* |  | 9.52 | 9.52 |
| LY | BNL3279-120 | 3 | 22.9 | 0.0014** |  |  |  |  |  |
| LY | BNL3479-240 | 13 | 132 | 0.0019** |  |  |  |  |  |
| LY | BNL3441-200 | 3 | 0 | 0.0065* |  |  |  |  |  |
| LY | BNL1721-200 | NaN | 31 | 0.0098* |  |  |  |  |  |
| LI | BNL3441-200 | 3 | 0 | 0.0041** |  |  |  |  |  |
| LY | JESPR220-161 | 20 | 29.2 |  | 0.0020** | 0.0019** |  | 11.92 | 12.11 |
| LY | JESPR220-171 | 20 | 29.2 |  | 0.0024** | 0.0023** |  | 11.52 | 11.66 |
| LY | BNL3452-170 | 19 | 18.9 |  | 0.0047** | 0.0041** |  | 10.06 | 10.41 |
| LY | BNL3452-200 | 19 | 18.9 |  | 0.0055* | 0.0052* |  | 9.71 | 9.87 |
| LI | BNL4108-180 | 6 | 139 |  | 0.0018** | 0.0033** |  | 15.40 | 13.06 |
| LI | BNL3423-240 | NaN | 45.5 |  | 0.0048** | 0.0051* |  | 13.31 | 12.19 |
| LI | BNL4108-163 | 6 | 139 |  | 0.0084* | 0.0081* |  | 12.17 | 11.22 |

**p* < 0.01; ***p* < 0.005, ****p* < 0.0005

^ linked to more than one trait

*r*^2^= phenotypic variance explained

**Table S7 Significant marker-trait associations in early sown 2019 environment using three different methods i.e. MLMM, CMLM and MLM**

| **Trait** | **SSR** | **Chromosome** | **Position** | ***p* value** | | | ***r*^2^ (PVE in% )** | | |
| --- | --- | --- | --- | --- | --- | --- | --- | --- | --- |
|  |  |  |  | **MLMM** | **CMLM** | **MLM** | **MLMM** | **CMLM** | **MLM** |
| DF | BNL3441-210 | 3 | 0 | 0.0015** |  |  |  |  |  |
| DF | BNL686-140 | 15 | 1 |  | 0.0083* | 0.0083* |  | 0.0841 | 0.0841 |
| PH | CGR5452-160 | 12 | 50 |  | 0.0039** |  |  | 0.1343 |  |
| PH | BNL1053-210 | 1 | 243 |  | 0.0075* | 0.0066* |  | 0.1207 | 0.1261 |
| PH | BNL3563-245 | 10 | 42.1 |  | 0.0093* |  |  | 0.1164 |  |
| PH | BNL3368-168 | NaN | 111 | 0.0088* |  |  |  |  |  |
| PH | BNL2572-230 | 4 | 92.7 | 0.0033** |  |  |  |  |  |
| BW | BNL1605-100 | 8 | 90 | 0.00145** |  |  |  |  |  |
| BW | CGR5452-160 | 12 | 50 | 0.00188** |  |  |  |  |  |
| BW | BNL2572-260 | 4 | 92.7 | 0.00381** |  |  |  |  |  |
| BW | BNL3976-165 | 7 | 186 | 0.00581* |  |  |  |  |  |
| BW | BNL1605-90 | 8 | 90 | 0.00669* |  |  |  |  |  |
| NB | BNL3279-120^ | 3 | 22.9 | 0.00084*** |  |  |  |  |  |
| NM | BNL4028-180 | 9 | 194 | 0.00349** |  |  |  |  |  |
| NM | BNL1693-240 | 15 | 124 | 0.00379** |  |  |  |  |  |
| NM | BNL686-140 | 15 | 1 | 0.00536* |  |  |  |  |  |
| NM | BNL1693-250 | 15 | 124 | 0.00660* |  |  |  |  |  |
| NM | BNL3280-220 | 18 | 26 |  | 0.00639* |  |  | 0.0959 |  |
| GOT | BNL2961-241 | 19 | 18.1 |  |  | 0.00980* |  |  | 0.1730 |
| SI | BNL3089-131 | 4 | 1.63 | 0.00299** |  |  |  |  |  |
| SI | BNL3089-141 | 4 | 1.63 | 0.00299** |  |  |  |  |  |
| SI | BNL448-216 | 20 | 17.9 | 0.00567* |  |  |  |  |  |
| SI | BNL3976-130 | 7 | 186 | 0.00629* |  |  |  |  |  |
| SI | BNL4108-163 | 6 | 139 |  |  | 0.00943* |  |  | 0.0959 |
| SCY/P | BNL3279-120^ | 3 | 22.9 | 0.00051*** |  |  |  |  |  |
| SCY/P | BNL4028-200^ | 9 | 194 |  | 0.00483** | 0.00689* |  | 0.1008 | 0.0892 |
| LY | BNL3279-120 | 3 | 22.9 | 0.0039** |  |  |  |  |  |
| LY | BNL3994-115 | 20 | 66 | 0.0071* |  |  |  |  |  |
| LY | BNL4028-200 | 9 | 194 |  | 0.0028** | 0.0043** |  | 0.1215 | 0.1154 |
| LI | BNL3594-236 | 10 | 229 | 0.0039** |  |  |  |  |  |
| LI | BNL3594-210 | 10 | 229 | 0.0053* |  |  |  |  |  |
| LI | BNL3902-200 | 15 | 14.6 | 0.0081* |  |  |  |  |  |

**p* <0.01; ***p* < 0.005, ****p* < 0.001

^ linked to more than one trait

*r*^2^= phenotypic variance explained

**Table S8 Significant marker-trait associations in normal sown 2018 environment using three different methods i.e. MLMM, CMLM and MLM**

| **Trait** | **SSR** | **Chromosome** | **Position** | ***p* value** | | | ***r*^2^ (PVE in %)** | | |
| --- | --- | --- | --- | --- | --- | --- | --- | --- | --- |
|  |  |  |  | **MLMM** | **CMLM** | **MLM** | **MLMM** | **CMLM** | **MLM** |
| DF | BNL3441-210 | 3 | 0 | 0.00149** |  |  |  |  |  |
| PH | BNL448-270 | 20 | 17.9 | 0.00249** | 0.00775* | 0.00829* |  |  | 0.13437 |
| DF | BNL686-140 | 15 | 1 | 0.00825* |  | 0.00825* |  |  | 0.08407 |
| NB | CGR5732-175^ | 19 | 130 | 0.00486** |  |  |  |  |  |
| NB | CGR5452-160 | 12 | 50 | 0.00720* |  |  |  |  |  |
| NB | BNL3479-240^ | 13 | 132 | 0.00875* |  |  |  |  |  |
| NB | BNL1721-200^ | 18 | 31 |  | 0.00177** | 0.00177** |  | 0.1359 | 0.1359 |
| NB | BNL3976-130 | 7 | 186 |  | 0.00795* | 0.00795* |  | 0.1040 | 0.1040 |
| NM | BNL3452-191 | 19 | 18.9 | 0.00459** |  |  |  |  |  |
| NM | BNL3257-230 | 8 | 162 | 0.00793* |  |  |  |  |  |
| NM | BNL3835-170 | 4 | 7.1 |  | 0.00800* |  |  | 0.1285 |  |
| GOT | BNL3452-200 | 19 | 18.9 | 0.00693* |  |  |  |  |  |
| GOT | BNL3368-155 | 26 | 111 |  | 0.00197** | 0.00182** |  | 0.1650 | 0.1694 |
| GOT | BNL3423-220 | 12 | 45.5 |  | 0.00331** | 0.00369** |  | 0.1542 | 0.1548 |
| GOT | BNL2570-260 | 20 | 141 |  | 0.00653* | 0.00784* |  | 0.1404 | 0.1396 |
| GOT | BNL1693-250 | 15 | 124 |  | 0.00722* | 0.00766* |  | 0.1384 | 0.1401 |
| GOT | BNL2921-150 | 1 | 46 |  | 0.00857* | 0.00645* |  | 0.1350 | 0.1435 |
| GOT | CGR5565-135 | 10 | 150 |  |  | 0.00908* |  |  | 0.1367 |
| SI | CGR5565-156 | 10 | 150 |  | 0.00724* | 0.00940* |  | 0.0999 | 0.0942 |
| SI | CGR5732-164 | 19 | 130 |  | 0.00996* |  |  | 0.0933 |  |
| SI | BNL2544-220 | 18 | 192 |  |  | 0.00811* |  |  | 0.0972 |
| SCY/P | BNL3479-240^ | 13 | 132 | 0.00035*** |  |  |  |  |  |
| SCY/P | CGR5732-175^ | 19 | 130 | 0.00115** |  |  |  |  |  |
| SCY/P | CGR5452-170 | 12 | 50 | 0.00990* |  |  |  |  |  |
| SCY/P | BNL1721-200^ | 18 | 31 |  | 0.00276** | 0.00276** |  | 0.1214 | 0.1214 |
| SCY/P | JESPR220-171 | 20 | 29.2 |  | 0.00448** | 0.00448** |  | 0.1110 | 0.1110 |
| SCY/P | NAU2355-258 | 6 | NA |  | 0.00533* | 0.00533* |  | 0.1074 | 0.1074 |
| SCY/P | NAU2355-250 | 6 | NA |  | 0.00533* | 0.00533* |  | 0.1074 | 0.1074 |
| SCY/P | BNL3479-250 | 13 | 132 |  | 0.00890* | 0.00890* |  | 0.0967 | 0.0967 |
| SCY/P | BNL3482-165 | 20 | 175 |  | 0.00985* | 0.00985* |  | 0.0946 | 0.0946 |
| LY | BNL3479-240 | 13 | 132 | 0.0003*** |  |  |  |  |  |
| LY | BNL686-165 | 15 | 1 | 0.0047** |  |  |  |  |  |
| LY | CGR5732-175 | 19 | 130 | 0.0058* |  |  |  |  |  |
| LY | BNL3436-210 | 10 | 29 | 0.0063* |  |  |  |  |  |
| LY | CGR5707-166 | 9 | 50 | 0.0080* |  |  |  |  |  |
| LY | CGR5707-176 | 9 | 50 | 0.0080* |  |  |  |  |  |
| LY | JESPR220-171 | 20 | 29.2 |  | 0.0021** | 0.0021** |  |  | 0.1265 |
| LY | BNL1721-200 | NaN | 31 |  | 0.0030** | 0.0030** |  |  | 0.1187 |
| LY | JESPR220-161 | 20 | 29.2 |  | 0.0033** | 0.0033** |  |  | 0.1169 |
| LY | NAU2355-250 | 6 | NA |  | 0.0052* | 0.0052* |  |  | 0.1071 |
| LY | NAU2355-258 | 6 | NA |  | 0.0052* | 0.0052* |  |  | 0.1071 |
| LI | BNL2544-220 | NaN | 192 |  | 0.0024** | 0.0013** |  |  | 0.1819 |
| LI | BNL3423-220 | NaN | 45.5 |  | 0.0029** | 0.0020** |  |  | 0.1722 |
| LI | CGR5565-135 | 10 | 150 |  | 0.0065* | 0.0083* |  |  | 0.1439 |
| LI | CGR5031-140 | 9 | 90 | 0.0057* |  |  |  |  |  |
| LI | BNL3441-200 | 3 | 0 | 0.0092* |  |  |  |  |  |

**p* < 0.01; ***p* < 0.005, *** *p* < 0.001

^ linked to more than one trait

*r*^2^= phenotypic variance explained

**Table S9 Significant marker-trait associations in normal sown 2019 environment using three different methods i.e. MLMM, CMLM and MLM**

| **Trait** | **SSR** | **Chromosome** | **Position** | ***p* value** | | | ***r*^2^ (PVE in %)** | | |
| --- | --- | --- | --- | --- | --- | --- | --- | --- | --- |
|  |  |  |  | **MLMM** | **CMLM** | **MLM** | **MLMM** | **CMLM** | **MLM** |
| DF | BNL3441-210 | 3 | 0 | 0.00149** |  |  |  |  |  |
| DF | BNL686-140 | 15 | 1 |  | 0.00825* | 0.00825* |  | 0.08408 | 0.08407 |
| PH | BNL1440-270 | 6 | 0 | 0.00003**** |  |  |  |  |  |
| PH | BNL1495-200 | 13 | 119 | 0.00099*** |  |  |  |  |  |
| PH | BNL1495-240 | 13 | 119 | 0.00309** |  |  |  |  |  |
| PH | BNL3994-125 | 20 | 66 | 0.00570* |  |  |  |  |  |
| PH | NAU2162-200 | 20 | 16 | 0.00918* |  |  |  |  |  |
| PH | NAU2162-210 | 20 | 16 | 0.00918* |  |  |  |  |  |
| PH | BNL1053-210 | 1 | 243 |  |  | 0.00888* |  |  | 0.11581 |
| BW | BNL1605-100 | 8 | 90 | 0.00302** |  |  |  |  |  |
| BW | BNL3976-165^ | 7 | 186 | 0.00414** |  |  |  |  |  |
| BW | BNL827-150 | 10 | 26.3 | 0.00987* |  |  |  |  |  |
| BW | NAU1141-210 | 18 | 100 |  | 0.00151** | 0.00151** |  | 0.1752 | 0.1752 |
| BW | NAU1141-220 | 18 | 100 |  | 0.00151** | 0.00151** |  | 0.1752 | 0.1752 |
| BW | BNL827-168 | 10 | 26.3 |  | 0.00266** | 0.00266** |  | 0.1634 | 0.1634 |
| BW | NAU1233-265 | 3 | 84.9 |  | 0.00948* | 0.00948* |  | 0.1379 | 0.1379 |
| BW | NAU1233-255 | 3 | 84.9 |  | 0.00948* | 0.00948* |  | 0.1379 | 0.1379 |
| NB | BNL2544-240 | 18 | 192 | 0.00538* |  |  |  |  |  |
| NB | BNL3257-230 | 8 | 162 | 0.00937* |  |  |  |  |  |
| NM | BNL1064-145 | 6 | 8 | 0.00392** |  |  |  |  |  |
| NM | BNL1064-155 | 6 | 8 | 0.00392** |  |  |  |  |  |
| NM | BNL3976-165^ | 7 | 186 | 0.00581* |  |  |  |  |  |
| NM | BNL1551-180 | 13 | 149 |  | 0.00362** | 0.00455** |  | 0.1556 | 0.1030 |
| NM | BNL3085-110^ | 1 | 98 |  | 0.00681* |  |  | 0.1423 |  |
| NM | BNL3085-120^ | 1 | 98 |  | 0.00681* |  |  | 0.1423 |  |
| NM | BNL3089-141 | 4 | 1.63 | 0.00645* |  | 0.00645* |  |  | 0.0956 |
| NM | BNL3089-131 | 4 | 1.63 | 0.00645* |  | 0.00645* |  |  | 0.0956 |
| NM | BNL3479-260 | 13 | 132 | 0.00820* |  | 0.00820* |  |  | 0.0905 |
| GOT | BNL1066-150 | 3 | 35.6 | 0.00191** |  |  |  |  |  |
| GOT | BNL2570-260 | 20 | 141 | 0.00498** |  |  |  |  |  |
| GOT | BNL686-165^ | 15 | 1 | 0.00730* |  |  |  |  |  |
| GOT | BNL3482-145 | 20 | 175 |  | 0.00599* | 0.00599* |  | 0.1085 | 0.1085 |
| GOT | CGR5193-160 | 12 | 110 |  | 0.00609* | 0.00609* |  | 0.1081 | 0.1081 |
| GOT | BNL4028-190 | 9 | 194 |  | 0.00627* | 0.00627* |  | 0.1075 | 0.1075 |
| SI | BNL2921-142 | 1 | 46 | 0.00479** |  |  |  |  |  |
| SI | BNL686-160 | 15 | 1 | 0.00765* |  |  |  |  |  |
| SI | NAU1167-197 | 2 | 11 | 0.00766* |  |  |  |  |  |
| SI | NAU1167-207 | 2 | 11 | 0.00766* |  |  |  |  |  |
| SI | CGR5534-141 | 2 | 90 |  | 0.00867* | 0.00808* |  | 0.1000 | 0.1024 |
| SI | CGR5534-150 | 2 | 90 |  | 0.00867* | 0.00808* |  | 0.1000 | 0.1024 |
| SCY/P | BNL686-165^ | 15 | 1 | 0.00654* |  |  |  |  |  |
| SCY/P | BNL3085-110^ | 1 | 98 |  | 0.00627* | 0.00627* |  | 0.1087 | 0.1087 |
| SCY/P | BNL3085-120^ | 1 | 98 |  | 0.00627* | 0.00627* |  | 0.1087 | 0.1087 |
| SCY/P | JESPR220-171 | 20 | 29.2 |  | 0.00680* | 0.00680* |  | 0.1070 | 0.1070 |
| LY | BNL1066-150 | 3 | 35.6 | 0.0051* |  |  |  |  |  |
| LY | BNL1551-170 | 13 | 149 | 0.0069* |  |  |  |  |  |
| LY | BNL946-239 | 20 | 77 |  | 0.0098* | 0.0098* |  | 0.0964 | 0.0964 |
| LI | BNL3994-115 | 20 | 66 | 0.0030** |  |  |  |  |  |
| LI | CGR5732-206 | 19 | 130 |  | 0.0019** | 0.0024** |  | 0.1400 | 0.1342 |

**p* < 0.01; ***p* < 0.005

^ linked to more than one trait

*r*^2^= phenotypic variance explained

**Table S10 Significant marker-trait associations in late sown 2018 environment using three different methods i.e. MLMM, CMLM and MLM**

| **Trait** | **SSR** | **Chromosome** | **Position** | ***p* value** | | | ***r*^2^ (PVE in %)** | | |
| --- | --- | --- | --- | --- | --- | --- | --- | --- | --- |
|  |  |  |  | **MLMM** | **CMLM** | **MLM** | **MLMM** | **CMLM** | **MLM** |
| DF | BNL3441-210 | 3 | 0 | 0.00149** |  |  |  |  |  |
| DF | BNL686-140 | 15 | 1 |  | 0.00825* | 0.00825* |  | 0.08408 | 0.08407 |
| PH | BNL3099-160 | 9 | 144 | 0.00832* |  |  |  |  |  |
| PH | BNL4028-180 | 9 | 194 | 0.00937* |  |  |  |  |  |
| PH | NAU2162-200 | 20 | 16 | 0.00980* |  |  |  |  |  |
| PH | NAU2162-210 | 20 | 16 | 0.00980* |  |  |  |  |  |
| PH | CGR5452-160 | 12 | 50 |  | 0.00977* | 0.00916* |  | 0.16104 | 0.15098 |
| BW | BNL1551-170 | 13 | 149 | 0.00758* |  |  |  |  |  |
| BW | BNL448-210 | 20 | 17.9 | 0.00990* |  |  |  |  |  |
| BW | BNL3502-160 | 5 | 139 |  | 0.00276** | 0.00276** |  | 0.2143 | 0.2143 |
| BW | BNL827-168 | 10 | 26.3 |  | 0.00543* | 0.00543* |  | 0.2014 | 0.2014 |
| BW | BNL946-230 | 20 | 77 |  | 0.00834* | 0.00834* |  | 0.1934 | 0.1934 |
| BW | BNL3359-220 | 6 | 6 |  | 0.00854* | 0.00854* |  | 0.1930 | 0.1930 |
| NB | BNL1681-100^ | 1 | 249 | 0.00310** |  |  |  |  |  |
| NB | BNL2921-132 | 1 | 46 | 0.00622* |  |  |  |  |  |
| NB | BNL3452-200 | 19 | 18.9 |  | 0.00675* | 0.00675* |  | 0.2318 | 0.2318 |
| NB | BNL1440-259 | 6 | 0 |  | 0.00996* | 0.00996* |  | 0.2249 | 0.2249 |
| NB | BNL1440-270 | 6 | 0 |  | 0.00996* | 0.00996* |  | 0.2249 | 0.2249 |
| NM | BNL2572-240 | 4 | 92.7 |  | 0.00274** | 0.00254** |  | 0.1188 | 0.1179 |
| NM | BNL4035-220 | 12 | 49 |  | 0.00468** | 0.00892* |  | 0.1073 | 0.0912 |
| NM | CGR5452-180 | 12 | 50 |  | 0.00493** | 0.00646* |  | 0.1062 | 0.0980 |
| NM | BNL4108-180^ | 6 | 139 |  | 0.00495** | 0.00806* |  | 0.1061 | 0.0933 |
| NM | CGR5193-175 | 12 | 110 |  | 0.00783* |  |  | 0.0965 |  |
| NM | BNL3594-210 | 10 | 229 |  | 0.00810* | 0.00508* |  | 0.0958 | 0.1030 |
| NM | BNL4108-170 | 6 | 139 |  | 0.00821* |  |  | 0.0955 |  |
| NM | CGR5161-128 | 8 | 110 |  |  | 0.00935* |  |  | 0.0903 |
| GOT | BNL1605-90 | 8 | 90 | 0.00140** |  |  |  |  |  |
| GOT | BNL4034-86 | 3 | 40 | 0.00977* |  |  |  |  |  |
| GOT | BNL3261-200 | 12 | 6.5 |  | 0.00133** | 0.00145** |  | 0.1337 | 0.1308 |
| GOT | BNL3998-190 | 19 | 210 |  | 0.00431** | 0.00548* |  | 0.1081 | 0.1020 |
| SI | BNL3261-220 | 12 | 6.5 | 0.00116** |  |  |  |  |  |
| SI | BNL1066-150 | 3 | 35.6 | 0.00271** |  |  |  |  |  |
| SI | CGR5732-175 | 19 | 130 | 0.00789* |  |  |  |  |  |
| SI | NAU1369-242 | 8 | 66.3 |  | 0.00281** | 0.00132** |  | 0.1621 | 0.1505 |
| SI | NAU1369-250 | 8 | 66.3 |  | 0.00281** | 0.00132** |  | 0.1621 | 0.1505 |
| SI | CGR5732-164 | 19 | 130 |  | 0.00684* | 0.00822* |  | 0.1440 | 0.1120 |
| SI | BNL4108-180^ | 6 | 139 |  | 0.00729* |  |  | 0.1428 |  |
| SI | BNL786-110 | 15 | 100.9 |  | 0.00806* | 0.00496** |  | 0.1408 | 0.1224 |
| SI | BNL1681-100^ | 1 | 249 |  | 0.00843* | 0.00269** |  | 0.1399 | 0.1352 |
| SI | BNL2590-190 | 15 | 211 |  | 0.00940* | 0.00442** |  | 0.1377 | 0.1248 |
| SI | BNL3452-180 | 19 | 18.9 |  | 0.00959* |  |  | 0.1373 |  |
| SI | BNL3976-130 | 7 | 186 |  |  | 0.00567* |  |  | 0.1196 |
| SCY/P | BNL3279-120 | 3 | 22.9 | 0.00113** |  |  |  |  |  |
| SCY/P | BNL1681-100 | 1 | 249 | 0.00398** |  |  |  |  |  |
| SCY/P | BNL3257-210 | 8 | 162 | 0.00576* |  |  |  |  |  |
| SCY/P | BNL3099-168 | 9 | 144 |  | 0.00608* | 0.00608* |  | 0.1025 | 0.1025 |
| LY | BNL3279-120 | 3 | 22.9 | 0.00180** |  |  |  |  |  |
| LY | BNL1681-100 | 1 | 249 | 0.00353** |  |  |  |  |  |
| LI | BNL3261-220 | 12 | 6.5 | 0.00351** |  |  |  |  |  |
| LI | BNL448-222 | 20 | 17.9 | 0.00926* |  |  |  |  |  |
| LI | BNL2590-190 | 15 | 211 |  | 0.00488** | 0.00636* |  | 0.15082 | 0.12533 |
| LI | BNL4108-180 | 6 | 139 |  | 0.00560* | 0.00766* |  | 0.14804 | 0.12153 |
| LI | CGR5161-117 | 8 | 110 |  | 0.00942* | 0.00781* |  | 0.13770 | 0.12115 |

**p* < 0.01; ***p* < 0.005;

^ linked to more than one trait

*r*^2^= phenotypic variance explained

**Table S11 Significant marker-trait associations in late sown 2019 environment using three different methods i.e. MLMM, CMLM and MLM**

| **Trait** | **SSR** | **Chromosome** | **Position** | ***p* value** | | | ***r*^2^ (PVE in %)** | | |
| --- | --- | --- | --- | --- | --- | --- | --- | --- | --- |
|  |  |  |  | **MLMM** | **CMLM** | **MLM** | **MLMM** | **CMLM** | **MLM** |
| DF | BNL3441-210 | 3 | 0 | 0.00149** |  |  |  |  |  |
| DF | BNL686-140 | 15 | 1 |  | 0.00825* | 0.00825* |  | 0.08408 | 0.08407 |
| PH | BNL3257-220 | 8 | 162 | 0.00048** |  |  |  |  |  |
| PH | BNL3502-150 | 5 | 139 | 0.00728* |  |  |  |  |  |
| PH | BNL1053-193 | 1 | 243 |  | 0.00947* |  |  | 0.15446 |  |
| PH | BNL3441-180 | 3 | 0 |  | 0.00981* | 0.00884* |  | 0.15378 | 0.15891 |
| BW | BNL3261-200 | 12 | 6.5 | 0.00546* |  |  |  |  |  |
| BW | BNL3976-165^ | 7 | 186 | 0.00590* |  |  |  |  |  |
| BW | BNL3441-210 | 3 | 0 | 0.00666* |  |  |  |  |  |
| BW | BNL3590-190 | 2 | 61.5 | 0.00876* |  |  |  |  |  |
| BW | BNL3085-110 | 1 | 98 |  | 0.00065*** | 0.00068*** |  | 0.1645 | 0.1583 |
| BW | BNL3085-120 | 1 | 98 |  | 0.00065*** | 0.00068*** |  | 0.1645 | 0.1583 |
| BW | BNL2572-260 | 4 | 92.7 |  | 0.00399** | 0.00339** |  | 0.1251 | 0.1233 |
| BW | BNL3479-250 | 13 | 132 |  | 0.00631* | 0.00569* |  | 0.1156 | 0.1124 |
| NB | BNL1066-140^ | 3 | 35.6 | 0.00395** |  |  |  |  |  |
| NB | BNL3257-230^ | 8 | 162 | 0.00400** |  |  |  |  |  |
| NB | BNL3368-168^ | 26 | 111 |  | 0.00544* | 0.00544* |  | 0.1035 | 0.1035 |
| NM | BNL3998-190 | 19 | 210 | 0.00465** |  |  |  |  |  |
| NM | BNL3482-145 | 20 | 175 | 0.00833* |  |  |  |  |  |
| NM | BNL409-100 | 20 | 91 |  | 0.00204** | 0.00204** |  | 0.1767 | 0.1767 |
| NM | BNL448-210 | 20 | 17.9 |  | 0.00241** | 0.00241** |  | 0.1733 | 0.1733 |
| GOT | BNL3099-160 | 9 | 144 |  | 0.00027**** | 0.00016**** |  | 0.1955 | 0.1924 |
| GOT | BNL1421-200 | 13 | 119 |  | 0.00961* | 0.00919* |  | 0.1194 | 0.1040 |
| GOT | BNL1122-184 | 13 | 47.4 |  |  | 0.00852* |  |  | 0.1056 |
| GOT | BNL1122-174 | 13 | 47.4 |  |  | 0.00921* |  |  | 0.1040 |
| SI | BNL4035-200 | 18 | 49 | 0.00138** |  |  |  |  |  |
| SI | BNL4035-220 | 18 | 49 | 0.00150** |  |  |  |  |  |
| SI | BNL1551-190 | 13 | 149 | 0.00784* |  |  |  |  |  |
| SI | BNL2590-200 | 15 | 211 | 0.00859* |  |  |  |  |  |
| SI | BNL686-165 | 15 | 1 |  | 0.00613* | 0.00429** |  | 0.2061 | 0.2056 |
| SI | BNL3976-145 | 7 | 186 |  | 0.00670* | 0.00581* |  | 0.2044 | 0.1998 |
| SCY/P | BNL3279-120 | 3 | 22.9 | 0.00422** |  |  |  |  |  |
| SCY/P | BNL3257-230^ | 8 | 162 | 0.00470** |  |  |  |  |  |
| SCY/P | BNL3257-210 | 8 | 162 | 0.00601* |  |  |  |  |  |
| SCY/P | BNL3976-165^ | 7 | 186 | 0.00769* |  |  |  |  |  |
| SCY/P | BNL2847-250 | 9 | 88.9 | 0.00881* |  |  |  |  |  |
| SCY/P | BNL1066-140^ | 3 | 35.6 | 0.00914* |  |  |  |  |  |
| SCY/P | BNL2847-260 | 9 | 88.9 |  | 0.00447** | 0.00447** |  | 0.1213 | 0.1213 |
| SCY/P | BNL3368-168^ | 18 | 111 |  | 0.00769* | 0.00769* |  | 0.1101 | 0.1101 |
| LY | BNL1066-140 | 3 | 35.6 | 0.00396** |  |  |  |  |  |
| LY | BNL3099-176 | 9 | 144 | 0.00405** |  |  |  |  |  |
| LY | BNL2847-250 | 9 | 88.9 | 0.00446** |  |  |  |  |  |
| LY | BNL3279-120 | 3 | 22.9 | 0.00752* |  |  |  |  |  |
| LY | BNL3368-168 | NaN | 111 |  | 0.00387** | 0.00387** |  | 0.13967 | 0.13967 |
| LI | BNL4035-200 | NaN | 49 | 0.00008***** |  |  |  |  |  |
| LI | BNL3482-165 | 20 | 175 |  | 0.00345** | 0.00195** |  | 0.17763 | 0.18480 |
| LI | BNL686-165 | 15 | 1 |  |  | 0.00805* |  |  | 0.15653 |
| LI | BNL2499-240 | 19 | 96 |  |  | 0.00907* |  |  | 0.15421 |

**p* < 0.01; ***p* < 0.005; ****p* < 0.001; *****p* < 0.0005, ***** *p* < 0.0001

^ Linked to more than one trait

*r*^2^= phenotypic variance explained


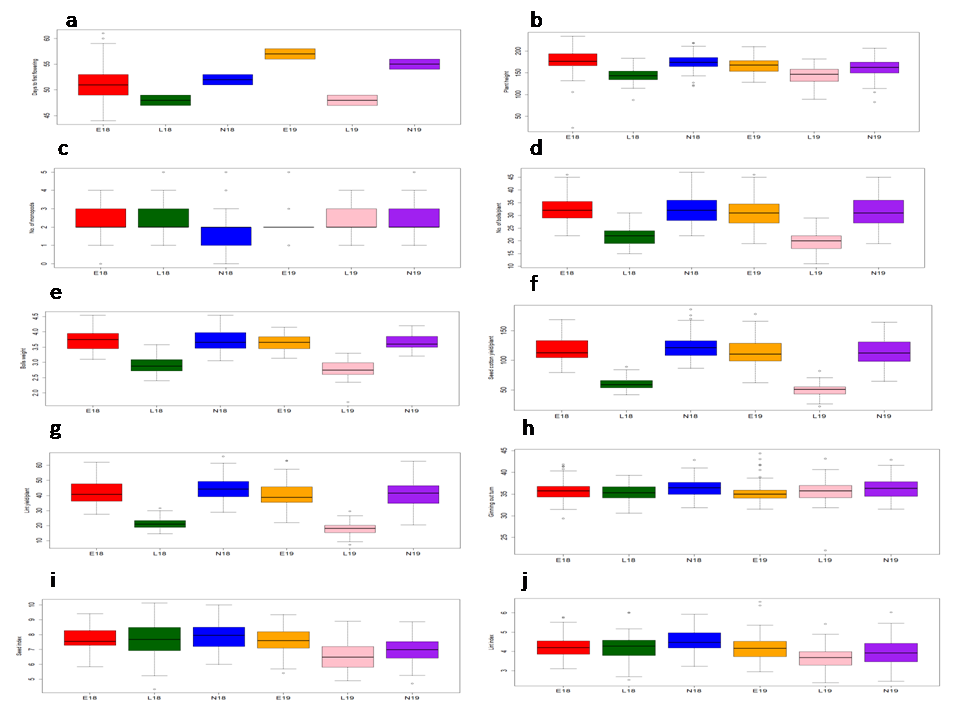


**Figure S1 Mean performance of trait among six environments. (a) days to first flower (b) plant height (c) number of monopods per plant (d) number of bolls per plant (e) boll weight among six environments (f) seed cotton yield per plant (g) lint yield per plant (h) ginning out turn (i) seed index (j) lint index among six environments**

**Figure S2 Graphical presentation of mean allelic pattern across the sub-populations**

**
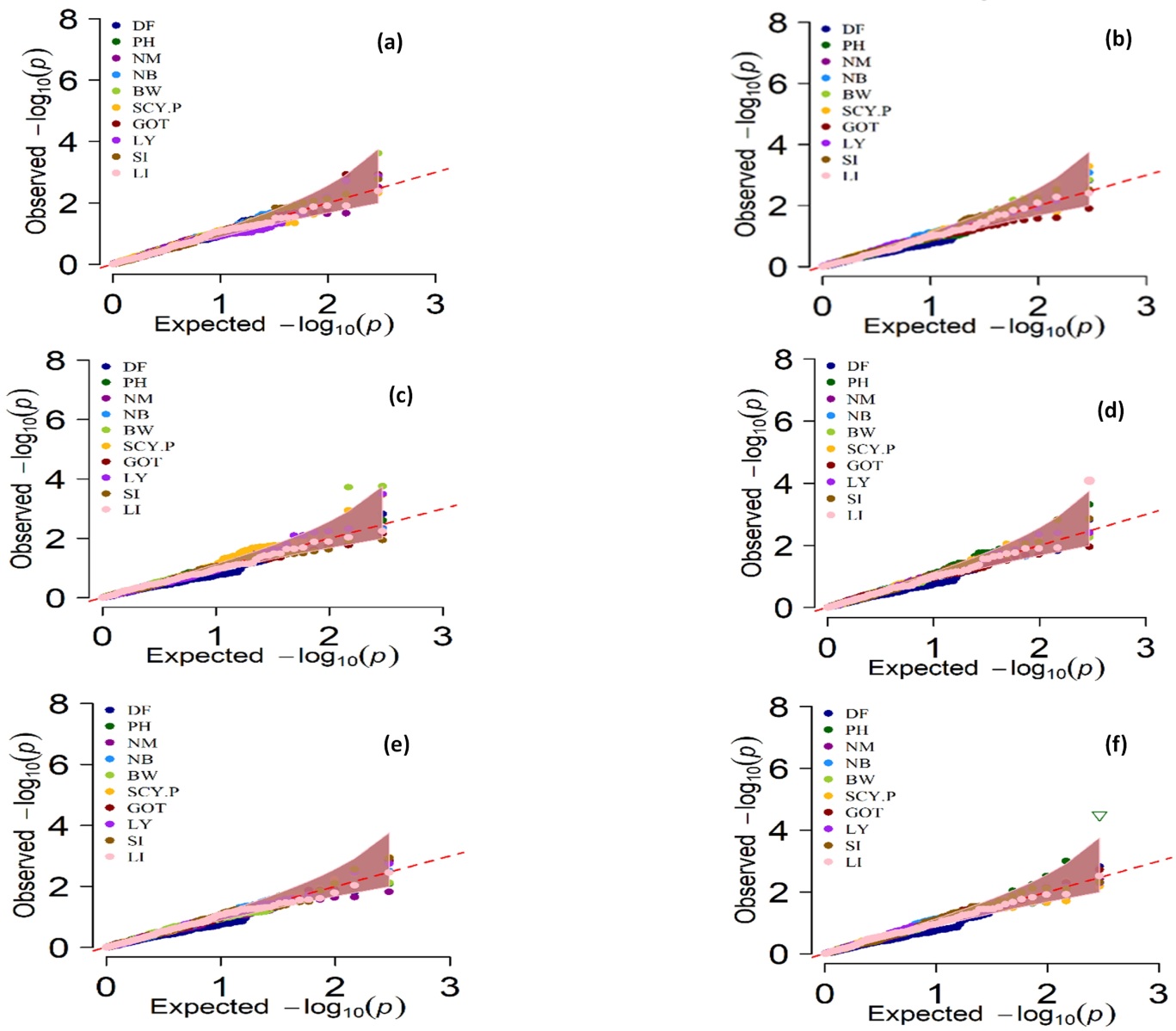
**

**Figure S3 Quantile-Quantile (QQ) plots between observed and expected p-values of association by MLMM model for all the traits used under study. (a) Early sown 2018 environment (b) early sown 2019 environment (c) normal sown 2018 environment (d) normal sown 2019 environment (e) late sown 2018 environment (f) late sown 2019 environment. DF= Days to flower, PH= Plant height, BW= Boll weight, NB= Number of boll per plant, NM= Number of monopods per plant, GOT= Ginning out turn, SI= Seed index, SCY/P= Seed cotton yield per plant, PH= Plant height, NM= Number of Monopods, NB= Number of Bolls per plant, BW= Boll weight, LY= Lint yield, LI= Lint index.**
